# PEDOTs in Bone Tissue Engineering Composites: Fabrication Strategies and Translational Hurdles

**DOI:** 10.34133/research.1394

**Published:** 2026-08-17

**Authors:** Meng Zhou, Wenhui Pei, Wenqi Shang, Guoyu Wu, Peng Wang, Qiangqiang Li, Mingguo Ma, Caoxing Huang

**Affiliations:** ^1^ Co-Innovation Center of Efficient Processing and Utilization of Forest Resources, Nanjing Forestry University, Nanjing 210037, China.; ^2^ Jiangxi Province Key Laboratory of Surface Engineering, School of Materials and Energy, Jiangxi Science and Technology Normal University, Nanchang 330013, China.; ^3^ State Key Laboratory of Pharmaceutical Biotechnology, Department of Sports Medicine and Adult Reconstructive Surgery, Nanjing Drum Tower Hospital, The Affiliated Hospital of Nanjing University Medical School, Nanjing 210008, China.; ^4^ Beijing Key Laboratory of Lignocellulosic Chemistry, College of Materials Science and Technology, Beijing Forestry University, Beijing, China.

## Abstract

Electroactive biomaterials represent a promising strategy for reconstructing the electrobiological microenvironment of bone and enhancing tissue regeneration. Among these materials, poly(3,4-ethylenedioxythiophene) (PEDOT) and its composites have attracted considerable attention because of their mixed electronic and ionic conductivity and compatibility with soft and porous scaffolds. However, existing reviews rarely address how fabrication strategies govern the relationships between structure, properties, and translational performance. This review establishes a fabrication, performance, and translation framework for PEDOT-based bone repair systems. Fabrication strategies are categorized into interfacial polymerization, bulk matrix and solution-processed conductive networks, patterned and fibrous conductive architectures, and porous and 3-dimensional scaffold fabrication and are correlated with conductive network topology, mechanical performance, and cytocompatibility. The mechanistic roles of PEDOT in osteogenesis, angiogenesis, immunomodulation, and electroresponsive drug release are further summarized. In addition, this review discusses the key trade-offs that limit practical applications, including the balance between conductivity and degradability, mechanical strength and porosity, as well as multifunctionality and manufacturability. Overall, this review provides a framework-oriented perspective to guide the rational design and clinical translation of PEDOT-based bioelectronic materials for bone tissue engineering.

## Introduction

In recent years, with the acceleration of global population aging and changes in lifestyle, the incidence of osteochondral defects has increased [1–3]. Global burden studies have shown that osteoarthritis affects more than 500 million people worldwide and is projected to become increasingly prevalent over the coming decades [4–6]. These disorders not only severely impair mobility and quality of life but also impose substantial economic and societal burdens on healthcare systems. As a highly dynamic and metabolically active organ, bone undergoes a natural repair process that involves complex cellular signaling pathways and microenvironmental regulation [7].

The complex pathological microenvironment represents a major obstacle to effective osteochondral repair [8]. When injuries involve extensive cartilage loss or severe fractures, they frequently exceed the intrinsic self-healing capacity of the body [5]. In particular, injuries accompanied by damage to major nerves and blood vessels further impair the regenerative process because of inadequate nutrient supply and disrupted signaling pathways [9]. Moreover, aging-associated declines in cellular regenerative capacity and reduced synovial fluid secretion further compromise tissue repair. Traditional autografts are limited by donor site availability and associated complications, whereas artificial implants often encounter challenges such as immune rejection, mechanical mismatch, and limited bioactivity [10,11].

**Fig. 1.**
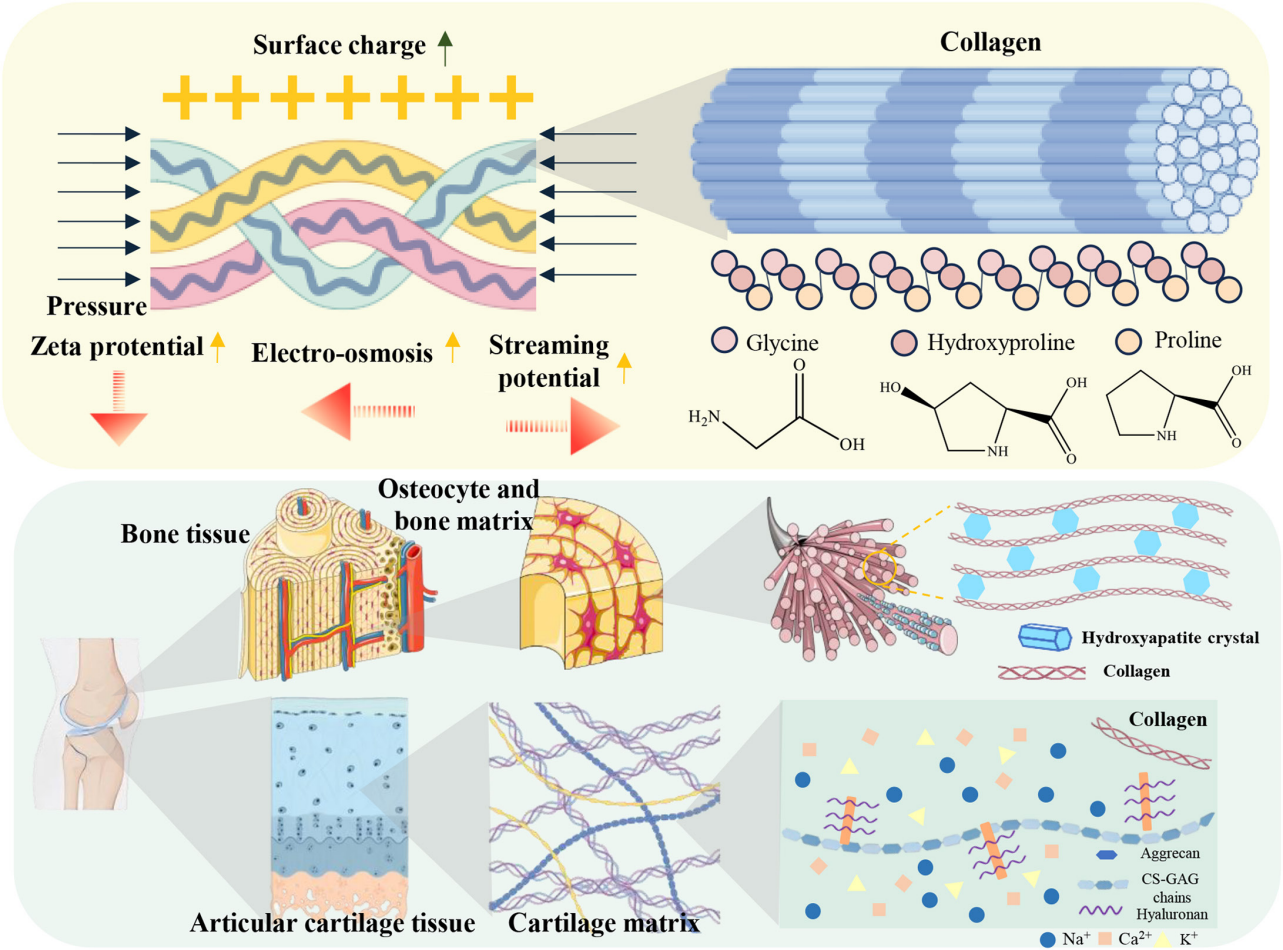
Diagram of the sources of bioelectricity in bones and joint cartilage.

To address these challenges, bone tissue engineering has emerged as a transformative strategy by mimicking the natural regenerative microenvironment [12,13]. Studies have demonstrated that endogenous bioelectrical signals play an indispensable role in bone and cartilage metabolism and repair. Bone tissue possesses intrinsic piezoelectric properties [14,15]. Under mechanical loading, such as walking or physical movement, deformation of collagen fibers generates transient piezoelectric potentials that precisely regulate the dynamic balance of bone remodeling (Fig. 1) [16]. Osteoblasts and chondrocytes activate downstream signaling pathways by sensing changes in transmembrane potential [17]. Therefore, actively reconstructing the endogenous electrobiological microenvironment of bone tissue using electroactive materials has become a major research focus for directing stem cell differentiation, promoting angiogenesis, and accelerating mineral deposition [12,14].

**Fig. 2.**
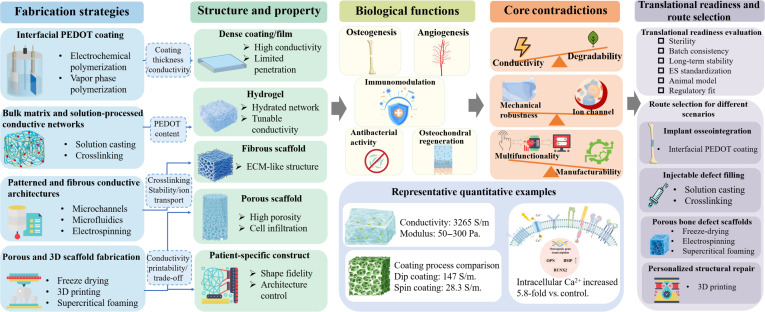
Framework for rational design and translation of poly(3,4-ethylenedioxythiophene) (PEDOTs) in bone tissue engineering.

As a new generation of electroactive materials, conductive polymers (CPs) have attracted considerable attention because of their ability to closely mimic the bioelectrical environment of native bone tissue [14,18]. Among the various CPs, poly(3,4-ethylenedioxythiophene) (PEDOT) and its composite poly(3,4-ethylenedioxythiophene):polystyrene sulfonate (PEDOT:PSS) have emerged as leading materials owing to their excellent electrical conductivity, superior biocompatibility, and mechanical flexibility [19–21]. Benefiting from their excellent solution processability, PEDOT and its composites (PEDOTs) can be fabricated into a variety of forms, including films, fibers, hydrogels, and aerogels, to satisfy the requirements of diverse clinical applications (Fig. 2) [21].

Recent reviews have summarized electroactive biomaterials for bone and cartilage regeneration, self-powered electrical stimulation (ES) systems, CPs for general biomedical applications, and 3-dimensional (3D) printed conductive or piezoelectric scaffolds [12–14,18,22–25]. Although these reviews provide comprehensive overviews of electroactive biomaterials, they generally consider PEDOT as only one representative CP and rarely examine how PEDOT-specific fabrication strategies determine conductive architecture, mechanical compatibility, cellular responses, sterilization tolerance, and translational potential. Therefore, this review focuses specifically on PEDOT by systematically analyzing its progression from material fabrication to biomedical application, rather than providing a general overview of electroactive biomaterials. The major contributions of this review are 3-fold. First, it systematically compares interfacial polymerization, bulk matrix and solution-processed conductive networks, patterned and fibrous conductive architectures, and porous and 3D scaffold fabrication with respect to electrical conductivity, mechanical performance, cytocompatibility, and manufacturability. Second, it elucidates how PEDOT-mediated electron and ion transport regulates the behavior of bone related cells and interacts with ES. Finally, it identifies the major bottlenecks that hinder clinical translation, including nondegradability, mismatched release kinetics of multiple bioactive factors, sterilization compatibility, batch-to-batch reproducibility, and the lack of standardized evaluation methods.

## The Properties of PEDOTs and Their Osseocompatibility

PEDOT, as a representative CP, has demonstrated significant potential in bone tissue engineering by evolving from a simple structural support material to a platform capable of precisely regulating the endogenous electrophysiological microenvironment [26,27]. Bone tissue is a complex system with intrinsic piezoelectric properties and self-regulatory capacity, and its repair process is highly dependent on electrical signal conduction [28,29]. The development of PEDOTs provides a biomimetic bridge for restoring the electrical homeostasis of damaged bone tissue.

### Conductivity

**Fig. 3.**
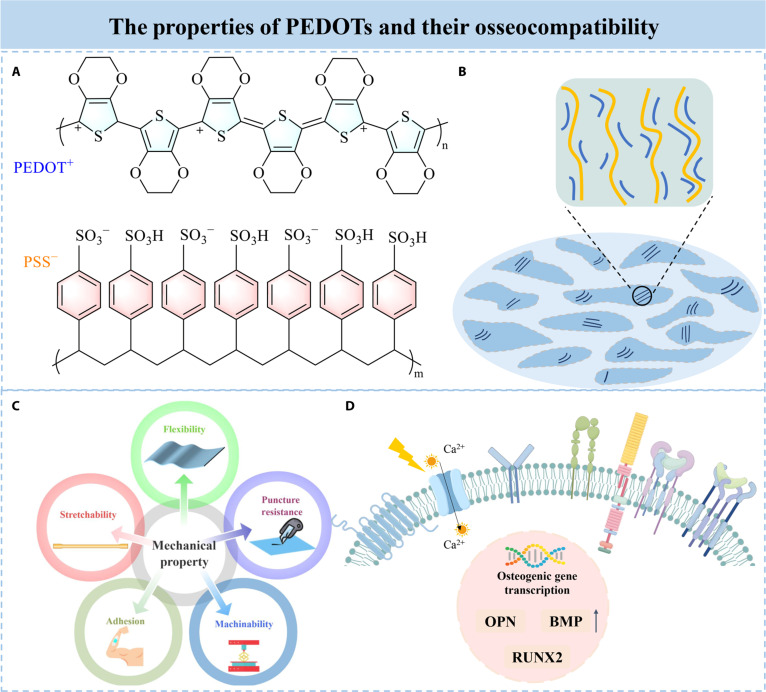
(A) The chemical structure of poly(3,4-ethylenedioxythiophene):polystyrene sulfonate (PEDOT:PSS) and (B) its microscopic structure in the dispersion solution. (C) Mechanical properties of PEDOTs and (D) the mechanism of osteogenesis promoted by electrical stimulation.

The repeating unit of PEDOT consists of thiophene rings containing alternating single and double bonds that form a continuous π-conjugated system, enabling extensive electron delocalization along the polymer backbone [30]. This molecular structure constitutes the physicochemical basis for its excellent electrical conductivity [31]. Human bone generates negative electrical potentials under mechanical loading, such as during walking. PEDOT:PSS, with a conductivity exceeding 10^3^ S/cm, can effectively conduct and amplify these weak electrophysiological signals, thereby compensating for the loss of electrical continuity at fracture sites [32,33]. PSS anions act as dopants that stabilize the oxidized state of PEDOT through electrostatic interactions, enabling the material to maintain stable electrical performance over prolonged periods, even in complex physiological environments such as fracture hematomas (Fig. 3A and B).

Existing studies suggest that PEDOT-mediated electrical cues primarily act through mixed electronic and ionic transport at the cell–material interface rather than through bulk conductivity alone [16]. Once a continuous conductive domain is established, ES can promote membrane depolarization, Ca^2+^ influx, and osteogenic signaling [17]. However, higher electrical conductivity does not necessarily correspond to superior bone regenerative performance, as cellular responses are also influenced by hydration, interfacial protein adsorption, matrix stiffness, pore accessibility, and degradation behavior. When bone tissue is damaged, its intrinsic electrical potential gradient is disrupted. Restoring this electrical activity is a critical factor in bone regeneration [34]. PEDOT can facilitate bone regeneration by mimicking or reconstructing this electrical microenvironment, thereby providing the electrostimulatory cues required for tissue repair. Electrical signals transmitted through conductive materials activate Ca^2+^ channels on the cell membrane, thereby promoting Ca^2+^ influx. For example, intracellular calcium concentrations in the calcium phosphate PEDOT:PSS-magnesium titanate-methacrylated alginate (CPM@MA) hydrogel with the ES group were 5.8-fold higher than those in the control group [35]. Ca^2+^ ions serve as central regulators of osteogenic differentiation and mineralization.

### Mechanical properties

In bone tissue engineering, the mechanical contribution of PEDOT-based systems should be interpreted according to the fabrication route [36,37]. For surface coatings and flexible films, PEDOT primarily enhances interfacial compliance, adhesion, and fatigue resistance, thereby reducing the mechanical mismatch at the implant–tissue interface (Fig. 3C). For example, flexible MXene-PEDOT/AgNWs electrodes with a 3D crosslinked network have demonstrated high tensile strain (up to 233%) and excellent fatigue resistance, highlighting the ability of PEDOT-containing networks to withstand repeated deformation [38]. However, such tensile properties or electrode-level performance should not be directly extrapolated to load-bearing bone scaffolds. For porous scaffolds or hydrogel-based bone substitutes, more relevant mechanical parameters include the wet-state compressive modulus, pore stability under cyclic loading, interfacial bonding strength, degradation-coupled mechanical retention, and conductivity retention during deformation. Therefore, the mechanical performance of PEDOT-based systems should not be evaluated solely on the basis of flexibility or stretchability. Instead, their mechanical properties should be tailored to the intended application, such as implant coatings, injectable fillers, osteochondral interface repair, or load-bearing bone defect reconstruction.

### Osseocompatibility

PEDOT has been demonstrated to exhibit excellent biocompatibility with no apparent cytotoxicity [39]. It can significantly enhance the adhesion, spreading, and proliferation of osteoblasts and bone mesenchymal stem cells (BMSCs). Electrical signals conducted through PEDOT, or the cellular responses induced by electric fields, activate voltage-gated calcium channels and mechanosensitive calcium channels on the cell membrane, resulting in extracellular Ca^2+^ influx (Fig. 3D) [40]. The elevated intracellular Ca^2+^ concentration subsequently activates signaling pathways such as the calmodulin/calcineurin/nuclear factor of activated T cells pathway, thereby inducing the expression of key osteogenic transcription factors, including runt-related transcription factor 2 (RUNX2), bone morphogenetic protein-2 (BMP-2), alkaline phosphatase (ALP), and others, ultimately promoting bone repair at the molecular level.

### The mechanism and advantages of PEDOT in electroactive biomaterials

Compared with polyaniline and polypyrrole, PEDOT:PSS generally exhibits superior aqueous processability, lower interfacial impedance, greater electrochemical stability, and better compatibility with soft hydrated matrices, making it particularly suitable for cell-contacting scaffolds and implant coatings [18–21]. Compared with carbon nanomaterials, MXenes, and metallic nanoparticles, PEDOT provides a softer polymeric interface while simultaneously serving as a conductive phase, dopant reservoir, and electroresponsive drug release layer. Compared with piezoelectric ceramics or poly(vinylidene fluoride)-based systems, PEDOT does not generate electrical signals under mechanical loading. Instead, it efficiently transmits externally applied or self-powered electrical cues within hydrated tissues. Therefore, the primary role of PEDOT in bone repair is not merely to increase electrical conductivity but to establish a mixed electronic and ionic interface that integrates ES with protein adsorption, ion exchange, drug release, and cellular signaling.

Mechanistically, PEDOTs promote bone regeneration through 4 interconnected pathways. First, continuous PEDOT-rich domains reduce charge-transfer resistance and facilitate the delivery of electrical cues to osteoblasts, BMSCs, chondrocytes, and macrophages. Second, ES mediated by PEDOT-based scaffolds regulates membrane potential and Ca^2+^ influx, thereby activating osteogenic signaling pathways involving ALP, RUNX2, osteocalcin (OCN), bone morphogenetic protein (BMP)/smad, and type I collagen (COL I) [12–17,35,40]. Third, PEDOT-based composites modulate the material–biological interface by altering hydrophilicity, protein adsorption, nanoscale surface topology, and the local release of ions such as Mg^2+^, Zn^2+^, Sr^2+^, and Ti species. Fourth, the intrinsic redox activity of PEDOT enables the electroresponsive release of anti-inflammatory or osteogenic molecules. However, this function must be coordinated with the sequential stages of bone healing.

## Fabrication Methods of PEDOTs for Bone Applications

To improve the logical consistency of fabrication classification, PEDOT-based systems are reorganized according to the spatial distribution of PEDOT and the topology of the resulting conductive network [21]. From this perspective, current fabrication strategies can be classified into 4 categories—interfacial PEDOT coating strategies, bulk matrix and solution-processed conductive networks, patterned and fibrous conductive architectures, and porous and 3D scaffold fabrication. This classification directly links fabrication routes with conductive network formation, mechanical and structural contributions, biological responses, and translational potential. Surface-confined coating strategies primarily enhance interfacial charge transfer and implant osseointegration. Bulk matrix strategies regulate mixed ionic and electronic transport within hydrated matrices while enabling drug and ion loading. Patterned and fibrous conductive architectures direct cellular organization and spatial signaling, whereas porous and 3D scaffold fabrication methods control bone ingrowth, vascularization, and defect-specific structural matching. Accordingly, Table 1 presents a cross-method comparison of the major PEDOT fabrication strategies, focusing on conductive network formation, structural contributions, cytocompatibility considerations, suitable application scenarios, and key limitations. Table 2 further summarizes representative PEDOT-based systems and correlates their electrical, mechanical, and biological properties with bone repair outcomes. Together, these tables establish a route-property-function framework for the rational selection of PEDOT-based systems for implant surface modification, injectable hydrogel repair, biomimetic fibrous scaffolds, vascularized porous constructs, patient-specific 3D scaffolds, and solvent-free foamed implants.

**Table 1.** The advantages and disadvantages of the preparation method for PEDOTs

Fabrication strategies	Method	Typical conductive network	Mechanical/structural contribution	Cell compatibility/risk	Best-fit scenarios	Main limitations
Interfacial PEDOT coating strategies	Electrochemical polymerization	1. Dense PEDOT coating 2. Substrate-grown network 3. High charge transfer	1. Thin surface layer 2. Limited mechanical support 3. Stable interfacial modification	1. Good if potential 2. Residual monomer risk	1. Metallic implant coatings 2. Titanium/ITO modification 3. Electrically triggered release	1. Conductive substrate required 2. Limited coating thickness 3. Poor 3D penetration
	Vapor phase polymerization	1. Conformal PEDOT layer 2. Surface-localized network 3. High local conductivity	1. Conformal surface modification 2. Pore architecture retention 3. Minimal scaffold deformation	1. Mild processing 2. Oxidant residue risk 3. Monomer removal needed	1. Porous surface modification 2. Functionalization 3. Implant interface coating	1. Diffusion gradient 2. Humidity sensitivity 3. Temperature dependence
Bulk matrix and solution-processed conductive networks	Solution casting	1. Dispersed PEDOT phase 2. Matrix-dependent network 3. Moderate conductivity	1. Flexible film formation 2. Tunable filler reinforcement 3. Membrane-like structure	1. Additive-friendly system 2. HA/Gel/alginate compatibility 3. Drug or ion loading	1. Bioactive membranes 2. Coating films 3. Drug-delivery films	1. Phase separation 2. Interrupted pathways 3. Insulating matrix dilution
	Crosslinking	1. Mixed ionic/electronic network 2. Hydrated conductive phase 3. Crosslink-dependent conductivity	1. Soft hydrogel network 2. Injectable structure 3. Tunable modulus	1. Cell encapsulation- friendly 2. High hydration 3. Crosslinker cytotoxicity risk	1. Minimally invasive gels 2. Cartilage-like defects 3. Nonload-bearing repair	1. Reduced chain mobility 2. Limited ion transport 3. Conductivity loss
Patterned and fibrous conductive architectures	Microchannel engineering	1. Conductive channel walls 2. Guided PEDOT pathways 3. Localized electrical interfaces	1. Perfusable channels 2. Improved mass transport 3. Spatial cell guidance	1. Enhanced cell migration 2. Improved nutrient exchange 3. Stiffness loss risk	1. Vascularized bone scaffolds 2. Neurovascularized repair 3. Thick conductive constructs	1. High fabrication precision 2. Channel collapse risk 3. Difficult electrical continuity
	Microfluidic fabrication	1. Patterned conductive domains 2. PEDOT:PSS microfibers/ microgels 3. Gradient conductive networks	1. Controlled microarchitecture 2. Core–shell structures 3. Localized electrical cues	1. Mild aqueous processing 2. Cell encapsulation potential 3. Shear/photoinitiator risk	1. Cell-laden hydrogels 2. Injectable microgels 3. Osteochondral gradients	1. Low throughput 2. Chip clogging 3. Scale-up difficulty
	Electrospinning	1. PEDOT-coated fibers 2. PEDOT-blended nanofibers 3. Percolated surface network	1. Biomimetic fibers 2. High surface area 3. Tensile guidance	1. Cell adhesion support 2. Cell alignment guidance 3. Solvent residue risk	1. Tendon–bone interface repair 2. Directional cell alignment 3. Localized drug delivery	1. Limited scalability 2. Weak compression strength 3. Limited through-thickness conductivity
Porous and 3D scaffold fabrication	Freeze drying	1. PEDOT on pore walls 2. Partially connected network 3. Low-to-medium conductivity	1. High porosity 2. Cancellous-like structure 3. Cell infiltration support	1. Gel/HA/PEDOT compatibility 2. Nutrient exchange support 3. Batch-dependent structure	1. Porous bone ingrowth 2. Vascularization scaffolds 3. Immunomodulatory cryogels	1. Random pore structure 2. Batch variability 3. Discontinuous conductivity
	3D printing	1. Programmable PEDOT pathways 2. Bioink-diluted network 3. Spatial conductive design	1. Patient-specific geometry 2. Controlled pore size 3. Gradient architecture	1. Bioink-dependent safety 2. Print fidelity concern 3. Posttreatment influence	1. Customized bone scaffolds 2. Osteochondral constructs 3. Smart circuit scaffolds	1. Conductivity– printability trade-off 2. Nozzle clogging 3. Postprocessing complexity
	Supercritical foaming	1. Isolated PEDOT domains 2. Partially connected network 3. Low conductivity	1. Solvent-free pores 2. Tunable porosity 3. Foam-like architecture	1. Low solvent residue 2. Polymer matrix- dependent biocompatibility 3. Degradation- dependent safety	1. Mild electroactive implants 2. Porous polymer foams	1. Conductive domain isolation 2. Limited electrical output 3. Poor pore interconnectivity

PEDOT:PSS, poly(3,4-ethylenedioxythiophene):polystyrene sulfonate; Gel, gelatin; BG, bioactive glass; HA, hyaluronic acid; 3D, 3-dimensional

**Table 2.** Comparison of the basic performances and possible applications of some typical PEDOTs with various morphology forms

Materials	Method/form	Conductivity (S cm^−1^; grade)	Structural	Biological function	Ref.
CPM@MA	Crosslinking/hydrogel	1.52 × 10^−3^ (medium)	Photopolymer hydrogel; ultimate load 35.6 MPa.	ES-triggered Ca^2+^ influx and osteogenic repair.	[35]
MXene-PEDOT:PSS/AgNWs	Crosslinking/film	/	3D crosslinked flexible electrode; tensile strain up to 233%.	Flexible electroactive interface with fatigue resistance.	[38]
ED-PEDOT	Electrochemical polymerization/film	2 × 10^4^ (high)	Dense and continuous conductive film.	Supports cell adhesion/proliferation and ES transmission.	[43]
NPEDOT/GAGs	Electrochemical polymerization/coating	/	GAG-guided nanostructured coating; reversible wettability.	Improves protein adsorption, MC3T3 adhesion, and osteogenic response.	[46]
rGO-PEDOT	Electrochemical polymerization/film	/	Microelectrode array with rGO topographic guidance.	hMSC alignment and electroresponsive DEX release; ALP up-regulated.	[47]
ZnP-PEDOT/TiO_2_	Electrochemical polymerization/coating	/	PEDOT/TiO_2_ nanotube coating; corrosion potential around −0.159 V.	Zn^2+^ release and electrochemical coupling promote osteogenesis.	[48]
PEDOT/BN-TiO_2_	Electrochemical polymerization/coating	/	Ceramic-reinforced conductive coating.	Improves cell proliferation on implant surfaces.	[49]
PEDOT/MWCNT	Vapor phase polymerization/film	/	Conformal PEDOT coating on CNT framework or 3D CNT/PEDOT network.	Improves hydrophilicity, stability, cell compatibility, and ES-guided osteogenesis.	[55]
PEG-PPG-PEG-PEDOT:Tos	Vapor phase polymerization/scaffold	/	3D electrically active coating while retaining scaffold pores.	Supports hMSC metabolic activity and morphology.	[56]
PEDOT:PSS/WPU/rGO	Solution casting/film	18.2 (high)	Highly flexible electrothermal film; elongation up to 530%.	Conformal interface for ES/thermal management.	[58]
HA/Gel/SA-(PEDOT PSS)	Solution casting/film	9.2 × 10^−3^ (medium)	Biomimetic HA/gelatin/alginate composite film.	Nearly 99% cell survival; drug-delivery potential.	[59]
PEDOT:PSS-coated PBI	Solution casting/coating	/	Bioactive conductive coating system.	Supports osteogenic repair.	[60]
PEDOT:PSS/PIn-5-NH_2_	Crosslinking/hydrogel	32.65 (high)	Interconnected 3D pores; elastic modulus 50–300 Pa.	Soft conductive matrix for cell migration and nutrient exchange.	[67]
PEDOT:PSS/MXene/GOPS	Freeze drying/scaffold	/	Porous sponge.	hASC osteogenic differentiation under ES.	[69]
PEDOT:PSS/PDMS	Microscale/coating	/	PEDOT:PSS microelectrode integrated with PDMS channel.	Efficient electroporation and cell-model construction.	[72]
PEDOT:PSS particles	Microscale/particle	/	Porous crystalline microcarriers with tunable porosity.	Enhances L929 proliferation and survival.	[73]
PEDOT/dopamine/PCL	Electrospinning/scaffold	/	Nanofibrous PCL/dopamine/PEDOT biomimetic matrix.	Tendon–bone repair; reduces muscle atrophy/fat accumulation.	[76]
Gel/BG/PEDOT:PSS	Freeze drying/scaffold	1.7 × 10^−4^ (low)	Porous crosslinked gelatin/bioactive-glass/PEDOT network.	Improves new bone formation and long-term scaffold support.	[79]
PEDOT:PSS/MWCNT/GOPS	Freeze drying/scaffold	/	Porous conductive scaffold	Osteogenic repair	[80]
Mg-GA MOFs/PEDOT:PSS	Freeze drying/scaffold	1.21 × 10^−3^ (medium)	100–200 μm cryogel pores; sustained Mg^2+^/GA release.	Anti-inflammatory, angiogenic and osteogenic repair.	[82]
Gel-PEDOT:PSS-CMBT	Freeze drying/scaffold	0.59 (medium)	Conductive-piezoelectric interconnection network.	Transmits local piezoelectric charge and activates osteogenic Ca^2+^ signaling.	[83]
PEDOT:PSS/PGS	3D Printing/scaffold	/	Hydrophobic microporous self-powered triboelectric scaffold.	Cartilage template formation and bone regeneration.	[88]
PLGA-PEDOT:PSS	Supercritical foaming/scaffold	1.0 × 10^−5^ (low)	Solvent-free porous scaffold; 50% porosity, pore size 30–200 nm.	Porous electroactive scaffold for electrically sensitive tissue repair.	[92]
Sr/Mg/hydroxyapatite/PEDOT:PSS	Electrochemical polymerization/coating	/	Bilayer interconnected porous coating.	Sr/Mg ion effects promote osseointegration and osteogenesis.	[96]
PEDOT:PSS/PCL/GOPS (DVS)	3D Printing/scaffold	/	Printed PCL scaffold with crosslinked PEDOT:PSS coating.	Osteogenic differentiation under ES and improved mineralization.	[100]
PEDOT-ACS	Freeze drying/scaffold	/	Freeze-dried cartilage-related scaffold.	Cartilage repair and chondrogenic matrix support.	[105]
PEDOT:PSS/GOPS	Freeze drying/scaffold	5.6 × 10^−6^ (low)	Crosslinked porous PEDOT:PSS scaffold.	Cartilage repair	[106]
PEDOT:PSS/COL I	Freeze drying/scaffold	/	Collagen-conducting polymer composite.	Osteogenic repair	[113]
PHA/PEDOT:PSS/Zn^2+^	Crosslinking/hydrogel	0.209 (medium)	Zn^2+^-containing conductive hydrogel.	Disease detection and electrochemical sensing.	[115]
PWH/PCL	3D Printing/scaffold	/	3D-printed scaffold with angiogenic/osteogenic design.	Osteogenic repair and angiogenesis.	[122]
PEDOT/Gel:HA:HAp	Freeze drying/scaffold	1.1 × 10^−6^ (low)	Porous conductive scaffold.	Osteogenic repair and angiogenesis.	[123]

PEDOT:PSS, poly(3,4-ethylenedioxythiophene):polystyrene sulfonate; ES, electrical stimulation; CPM@MA, calcium phosphate PEDOT:PSS-magnesium titanate-methacrylated alginate; ALP, alkaline phosphatase; COL I, type I collagen; ED-PEDOT, electrochemically deposited PEDOT; GAG, glycosaminoglycan; rGO, reduced graphene oxide; MWCNT, multiwalled carbon nanotube; PEG-PPG-PEG, poly(ethylene glycol)-block-poly(propylene glycol)-block-poly(ethylene glycol); WPU, waterborne polyurethane; Gel, gelatin; SA, sodium alginate; GOPS, glycidyloxypropyl trimethoxysilane; PDMS, polydimethylsiloxane; PCL, polycaprolactone; PGS, poly(glycerol sebacate); PLGA, poly(lactic-co-glycolic acid); hASC, human adipose-derived stem cell; DVS, divinyl sulfone; Mg-GA MOFs, Mg-gallic acid metal–organic frameworks; CMBT, calcium-manganese-doped barium titanate; BG, bioactive glass; PHA, poly(N-hydroxyethyl acrylamide-co-2-acrylamido-2-methyl-1-propanesulfonic acid); HA, hyaluronic acid; ITO, indium tin oxide; PWH, piezoelectric whitlockite; NPEDOT, nanostructured PEDOT; MC3T3, mouse embryonic osteoblasts cells; hMSCs, human mesenchymal stem cells; DEX, dexamethasone

### Interfacial PEDOT coating strategies

#### Electrochemical polymerization

Electrochemical polymerization is not only a widely used method for synthesizing PEDOT but also a core interfacial engineering strategy for the surface modification of bone implants [20,21]. Its primary advantage lies in enabling the in situ growth of PEDOT on bone implant surfaces with complex geometries through the adjustment of current, voltage, or monomer concentration [41,42]. The principal function of electrochemically polymerized PEDOT is not to provide bulk mechanical reinforcement but to establish a surface-confined conductive layer that promotes protein adsorption, early cell attachment, electrochemical coupling, and localized ES at the implant surface.

**Fig. 4.**
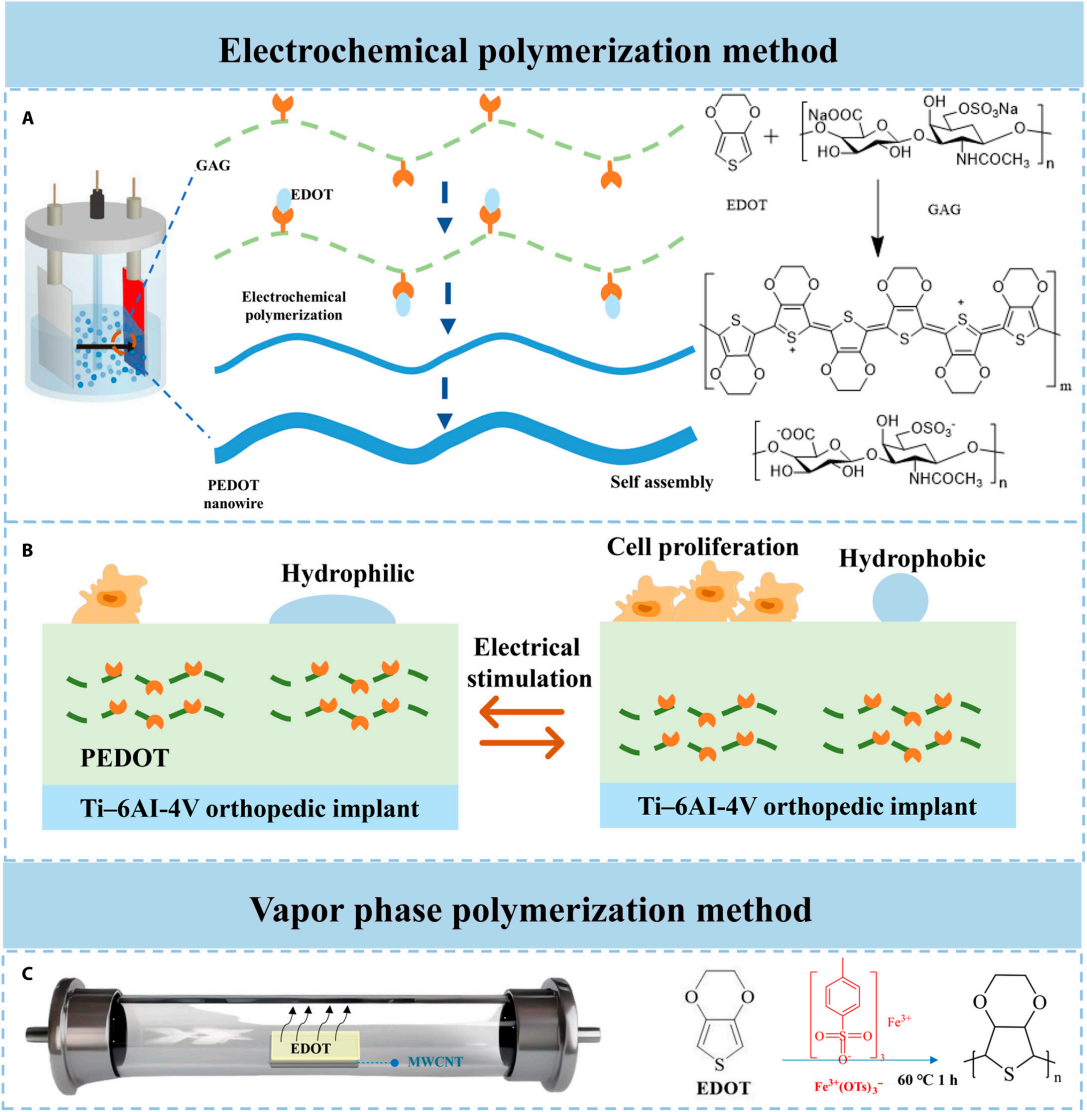
Representative electrochemical polymerization-based interfacial strategies for poly(3,4-ethylenedioxythiophene) (PEDOT) coatings, including (A) biomolecule-guided nanostructure formation and (B) wettability-regulated osteogenic interfaces. (C) The schematic diagram of PEDOT/multiwalled carbon nanotube (MWCNT) composite flakes via vapor phase polymerization method.

In reported studies on the electrochemical polymerization of PEDOT, 3 major design strategies have been employed. The first strategy is parameter-controlled PEDOT deposition, in which deposition conditions determine coating continuity, interfacial conductivity, and surface micro/nanotopography. Dense electrochemically deposited PEDOT films, for example, demonstrate that electrochemical deposition can produce continuous conductive coatings that support cell adhesion, proliferation, and electrical signal transmission [43]. The second strategy is biomolecule-assisted deposition. Glycosaminoglycans [44], collagen-like components, and other extracellular matrix (ECM)-derived molecules [45] can serve as molecular anchors or dopants during 3,4-ethylenedioxythiophene polymerization, thereby guiding the formation of nanostructured PEDOT coatings and improving the biological interface through enhanced wettability, protein adsorption, and osteoblast adhesion (Fig. 4A and B) [46]. The third strategy is composite interfacial deposition, in which carbon-based materials, metal oxides, bioceramics, or ion-releasing components are cointegrated with PEDOT to compensate for the limited mechanical stability and osteoinductive activity of pristine PEDOT. In these systems, PEDOT provides electrochemical coupling, whereas reduced graphene oxide (rGO) [47], TiO_2_ [48], BN [49], and other inorganic components contribute topographical guidance, corrosion resistance, mechanical reinforcement, or osteogenic ion release.

From a translational perspective, electrochemical polymerization should be regarded primarily as an implant interface engineering strategy. Its most suitable applications include titanium or other conductive implant coatings, enhancement of local osseointegration, and electroresponsive release from implant surfaces [50]. However, these advantages are accompanied by several limitations. The process generally requires a conductive substrate, and the deposited PEDOT layer is typically confined to the accessible surface [51,52]. Consequently, coating penetration into thick, tortuous, or highly porous 3D scaffolds remains limited. In addition, long-term coating adhesion, fatigue resistance under cyclic mechanical loading, sterilization compatibility, and stability in physiological fluids must be thoroughly validated before clinical translation. Therefore, electrochemical polymerization is highly valuable for surface bioelectronic functionalization but should not be regarded as a universal strategy for load-bearing or large-volume bone defect scaffolds.

#### Vapor phase polymerization

Vapor phase polymerization (VPP) is an advanced technique that involves the in situ polymerization of gaseous monomers on the surface of a substrate precoated with an oxidant [53]. Compared with liquid-phase polymerization, VPP overcomes the limitations associated with liquid wetting and enables conformal nanoscale coating of complex and porous substrates, thereby providing opportunities for constructing electroactive bone repair platforms with high structural stability [54]. Accordingly, its primary contribution to bone tissue engineering is not the formation of bulk matrices but the functionalization of pore walls or scaffold surfaces through the deposition of thin conductive PEDOT layers.

The structure–property relationship of VPP-derived PEDOT coatings is governed by several processing parameters. Monomer vapor pressure, oxidant loading, monomer-to-oxidant ratio, humidity, temperature, reaction time, and pore tortuosity collectively determine coating thickness, coating continuity, penetration depth, and local interfacial conductivity. Representative VPP-derived PEDOT systems illustrate this surface functionalization capability. For example, PEDOT coatings deposited on multiwalled carbon nanotube (MWCNT)-based substrates [55] (Fig. 4C) and poly(ethylene glycol)-block-poly(propylene glycol)-block-poly(ethylene glycol) (PEG-PPG-PEG)-PEDOT:Tos 3D scaffolds [56] demonstrate that VPP enhances local electroactivity and improves cytocompatibility without substantially disrupting the preexisting scaffold architecture.

The VPP process does not require the use of large quantities of liquid organic solvents [21,50]. Only simple washing is required to remove residual monomers and catalysts, thereby significantly reducing the risk of biological toxicity associated with solvent residues. This processing characteristic provides a distinct advantage for clinical implant applications that demand high levels of biosafety. Nevertheless, VPP still faces challenges associated with limited monomer vapor pressure and restricted diffusion within complex pore structures, which may result in nonuniform coating thickness or incomplete internal coating coverage. In the future, combining VPP with atomic layer deposition or intelligently designed responsive monomers is expected to enable more precise electrical functionalization of complex 3D microarchitectures.

### Bulk matrix and solution-processed conductive networks

#### Solution casting

The solution casting method is an indispensable and versatile platform for the fabrication of functional film materials [57]. Unlike interfacial polymerization, which primarily forms surface-confined PEDOT coatings, solution casting distributes PEDOT or PEDOT-containing components throughout a polymeric matrix and enables the incorporation of flexible polymers, inorganic fillers, natural biomacromolecules, ions, or therapeutic agents. Therefore, its value in bone tissue engineering lies not in the precise control of 3D architecture but in the cost-effective integration of electrical conductivity, mechanical flexibility, biological activity, and drug or ion loading capability within film-like or membrane-like constructs.

Across solution-cast PEDOT systems, the key structure–property relationship is governed by the balance between conductive network percolation and matrix dilution. Increasing the content of PEDOT or conductive fillers enhances the formation of conductive pathways, whereas excessive insulating matrix content, poor filler dispersion, or phase separation may disrupt conductive continuity [24]. Flexible matrices such as waterborne polyurethane improve deformability and interfacial compliance, whereas fillers such as rGO enhance electrical and thermal transport. Natural components, including hyaluronic acid (HA), gelatin (Gel), collagen, and sodium alginate (SA), compensate for the limited biological recognition motifs of PEDOT by improving hydrophilicity, protein adsorption, and cytocompatibility [21]. Consequently, the biological performance of solution-cast PEDOT composites is generally determined by the combined effects of conductive network formation and matrix-derived bioactivity rather than by PEDOT conductivity alone.

**Fig. 5.**
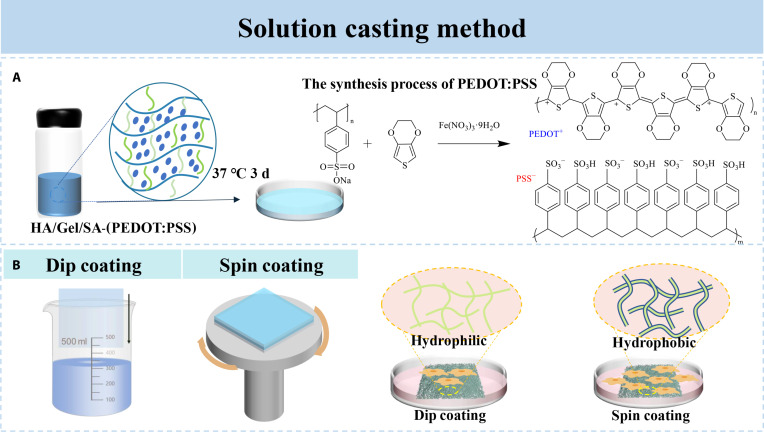
The schematic diagram of (A) hyaluronic acid/gelatin/sodium alginate-(poly(3,4-ethylenedioxythiophene):polystyrene sulfonate) [HA/Gel/SA-(PEDOT:PSS)] composite polymer film via solution casting method. (B) The effects of PEDOT:PSS-coated poly [2,2’-m-(phenylene)-5,5’-bibenzimidazole] (PBI) electrospun scaffolds prepared by dip-coating and spin-coating on cell proliferation.

The processing technique further influences the balance between conductivity and biological performance. For example, PEDOT/waterborne polyurethane/rGO films combine high flexibility, with a fracture elongation of 530%, and stable electrical conductivity (18.2 S/cm), highlighting their potential as conformal electroactive interfaces [58]. Similarly, HA/Gel/SA-PEDOT films demonstrate that natural bone-related components can improve cytocompatibility while maintaining moderate electrical conductivity (Fig. 5A) [59]. More importantly, comparisons of coating methods have shown that dip coating yields substantially higher electrical conductivity (147 S/m) than spin coating (28.3 S/m), whereas spin-coated surfaces may exhibit superior hydrophilicity and enhanced cell adhesion (Fig. 5B) [60]. These findings indicate that maximizing electrical conductivity should not be considered the sole design objective. For bone-related membranes and coatings, surface wettability, cytocompatibility, and interfacial stability should be optimized together with electrical performance.

Solution casting is particularly suitable for the fabrication of bioactive membranes, flexible coatings, electroactive films, and drug- or ion-delivery layers. Its major advantages include operational simplicity, low cost, compatibility with a wide range of additives, and facile modulation of film composition [61]. However, it also has several limitations, including phase separation, batch-to-batch variability, disruption of conductive pathways, limited control over pore architecture, and poor suitability for patient-specific or load-bearing 3D scaffolds. Therefore, solution casting should be regarded as a fabrication strategy for soft matrices and membrane-based systems rather than as a universal approach for complex bone defect reconstruction.

#### Crosslinking

**Fig. 6.**
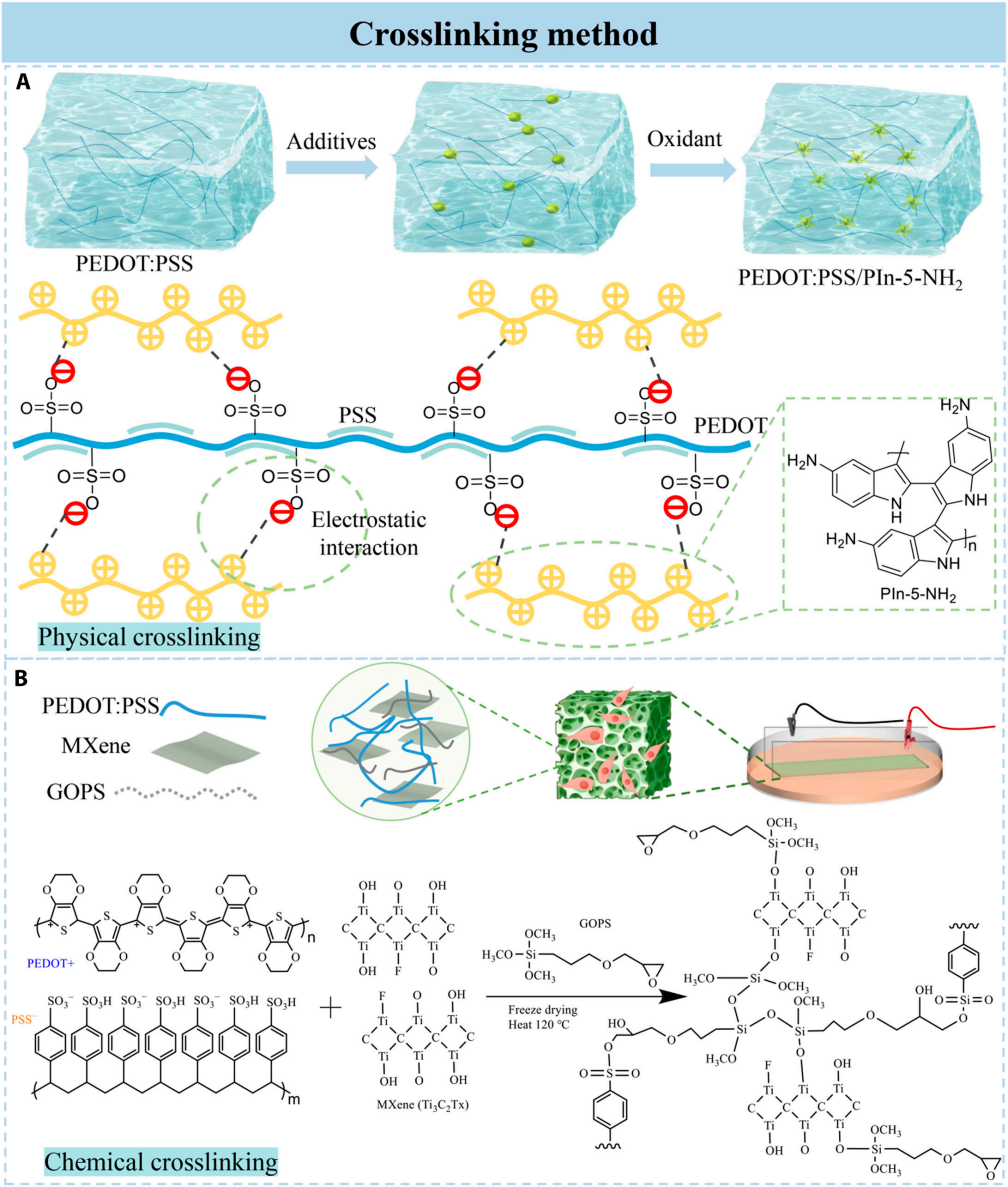
The preparation schematic of (A) poly(3,4-ethylenedioxythiophene):polystyrene sulfonate (PEDOT:PSS)/PIn-5-NH_2_ hydrogel and (B) PEDOT:PSS/MXene/glycidyloxypropyl trimethoxysilane (GOPS) composite scaffolds via crosslinking method.

Crosslinking serves as a fundamental architectural strategy for regulating the structural, physicochemical, and biological properties of PEDOT networks [62]. It is essential for maintaining the structural integrity of CPs in the complex physiological environment of bone tissue [63]. By carefully selecting the crosslinking mechanism, dynamic coordination among the mechanical modulus, degradation kinetics, and electrochemical activity of scaffolds can be achieved. Physical crosslinking relies on noncovalent interactions, including ionic bonding, hydrogen bonding, π-π stacking, and polymer chain entanglement, to construct the network [64–66]. Its greatest advantage is that it avoids the introduction of potentially cytotoxic chemical reagents, thereby significantly improving the biocompatibility of the material. The use of electrostatic interactions between positively charged molecules and negatively charged PSS chains is a common strategy for fabricating conductive hydrogels. For example, the positively charged polyindole derivative (PIn-5-NH_2_) can rapidly form a physically crosslinked PEDOT:PSS hydrogel with an electrical conductivity of up to 3265 S/m at room temperature (Fig. 6A) [67]. However, the relatively low mechanical modulus and reversible nature of physically crosslinked networks indicate that these systems are more suitable for injectable, cartilage-like, or nonload-bearing repair applications than for load-bearing bone reconstruction.

Compared with physical crosslinking, chemical crosslinking substantially enhances the long-term stability of materials in physiological fluids by forming stable covalent bonds [68]. The incorporation of silane coupling agents, such as 3-glycidyloxypropyl trimethoxysilane (GOPS), not only improves the barrier properties of the material but also enables the formation of siloxane bonds that enhance surface hydrophilicity, thereby providing a stable chemical interface for the long-term colonization of bone cells (Fig. 6B) [69]. In bone-related hydrogels, this strategy supports sustained ES, ion or drug loading, and localized osteogenic regulation. Nevertheless, excessive crosslinking may restrict PEDOT chain mobility, impede ionic transport, and reduce effective electrical conductivity because of increased network density. In addition, residual crosslinking agents and photoinitiators should be carefully evaluated for potential cytotoxicity when cells are encapsulated or when the material is intended for implantation.

Therefore, crosslinked PEDOT hydrogels should be evaluated using a framework that integrates structural stability, transport properties, and biological activity, rather than considering electrical conductivity or mechanical modulus alone. A moderate crosslinking density is required to maintain hydrogel integrity while preserving mixed ionic and electronic conduction and adequate pore accessibility [70]. From the perspective of clinical translation, crosslinking is particularly suitable for the fabrication of minimally invasive conductive hydrogels, soft tissue defect fillers, electroresponsive drug or ion delivery systems, and osteochondral interface repair materials. However, for large or load-bearing bone defects, crosslinked PEDOT hydrogels generally require additional structural reinforcement, mineral components, or porous scaffold support to achieve sufficient mechanical stability and long-term functional performance.

### Patterned and fibrous conductive architectures

#### Microchannel engineering

**Fig. 7.**
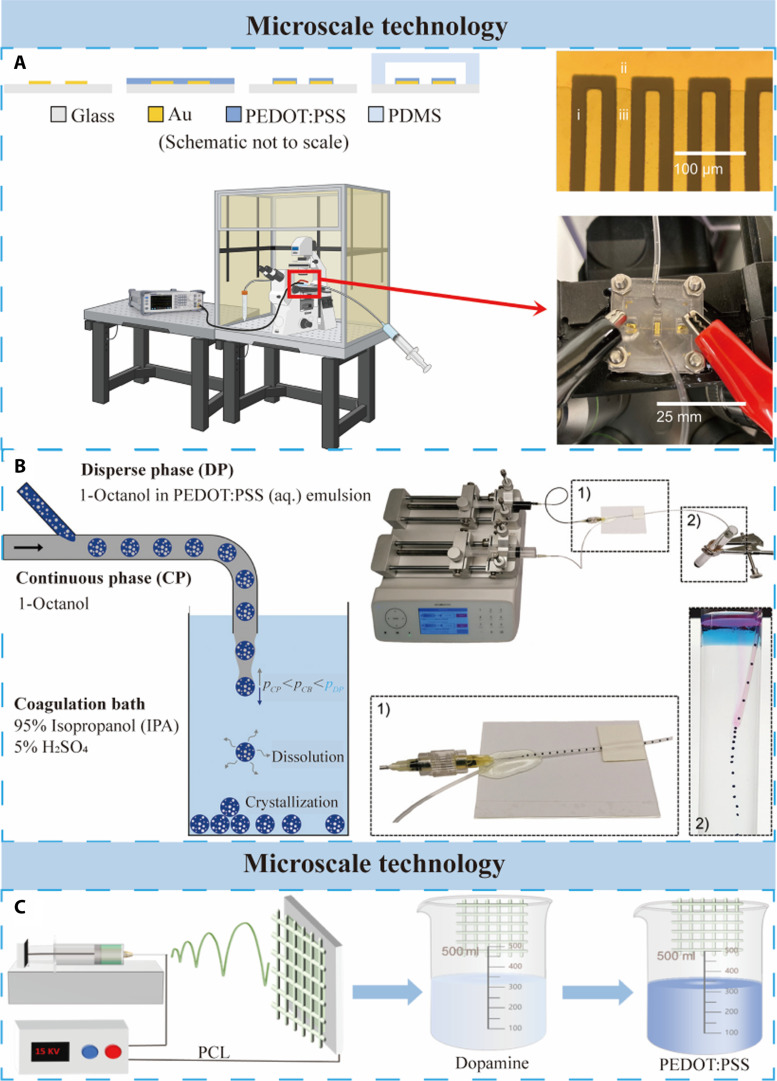
The process diagram of (A) poly(3,4-ethylenedioxythiophene):polystyrene sulfonate (PEDOT:PSS) electrodes [72], (B) porous PEDOT:PSS particles [73] prepared by microscale technology method, and (C) PEDOT/dopamine/polycaprolactone (PCL) nanofibers via electrospinning technology.

The integration of microscale engineering technologies, including microfluidics and microfabrication, with the electroactive polymer PEDOT:PSS has enabled the development of biomimetic integrated systems with highly organized architectures [71]. These systems are designed to simulate the complex physiological microenvironment of bone, thereby overcoming the limitations of conventional biological technologies, such as low precision in cellular manipulation, poor transfection efficiency, and limited industrial scalability. Consequently, they provide advanced platforms for investigating the mechanisms of bone diseases and developing precision therapeutic strategies. For example, Gharia et al. [72] fabricated a gold finger-shaped microelectrode array on a glass substrate using microfabrication techniques and subsequently deposited multilayer PEDOT:PSS films through spin coating and etching processes. The resulting structure was integrated into polydimethylsiloxane microfluidic channels (Fig. 7A). Benefiting from the excellent electrochemical activity and low interfacial impedance of PEDOT:PSS, this platform achieved highly efficient electroporation-mediated transfection of mouse bone marrow cells and successfully induced their differentiation into macrophages. Moreover, the transfected cells maintained normal morphology while exhibiting significantly higher fluorescence expression than that achieved using conventional methods. These findings provide a highly active cellular platform for precision treatment of bone tumors and studies of bone regeneration mechanisms.

#### Microfluidics fabrication

To achieve large-scale expansion and customized culture of bone repair cells, the development of highly biomimetic cellular microcarriers has become a major research focus. By combining microfluidic coflow technology with the nonsolvent-induced phase separation method, the high-throughput and continuous fabrication of porous PEDOT:PSS microparticles has been achieved (Fig. 7B) [73]. These highly crystalline PEDOT:PSS microparticles not only better mimic the physicochemical microenvironment of native bone tissue but also significantly enhance the proliferation and survival of L929 cells. This microfabrication strategy enables precise regulation of microparticle porosity and crystallinity, thereby overcoming the limited customizability and poor physiological relevance of conventional microcarriers and providing a foundation for constructing high-fidelity bone disease models [74].

At present, micro- and nanoscale engineering provides greater value as a platform for spatial regulation than as a clinically validated strategy for bone scaffold fabrication. Microchannel and microfluidic systems can organize conductive domains, improve nutrient transport, and generate cell-guiding architectures. However, most current evidence remains limited to in vitro cell manipulation, microcarrier fabrication, or disease modeling platforms. For bone regeneration, a critical unresolved question is whether these microscale conductive pathways can maintain electrical continuity, mechanical stability, and perfusability following implantation. Therefore, future studies should evaluate not only cell viability and transfection efficiency but also channel patency, vascular integration, and conductivity retention in thick bone-regenerative constructs.

#### Electrospinning technology

Electrospinning has become a core technique for constructing PEDOT-based biomimetic conductive materials because of its ability to precisely control nanofibrous architectures [75]. Polycaprolactone (PCL) nanofibers prepared by electrospinning provide a stable substrate for the subsequent deposition of dopamine and the PEDOT:PSS conductive layer (Fig. 7C) [76]. Their intrinsic nanoscale fibrous architecture imparts tissue-mimicking mechanical properties to the material. Combined with the electroactivity of PEDOT:PSS, these scaffolds form a bioactive matrix that integrates structural biomimicry with functional regulation.

Electrospun PEDOT-based scaffolds should be evaluated primarily according to their ability to provide anisotropic guidance rather than electrical conductivity alone. Compared with dense coatings or bulk hydrogels, electrospun matrices more closely reproduce the ECM-like fibrous topology and effectively guide cell alignment, migration, and interfacial tissue organization [77]. Consequently, they are more suitable for tendon–bone interface and osteochondral interface repair than for the reconstruction of large load-bearing bone defects. Nevertheless, limited through-thickness electrical conductivity, low compressive strength, and residual solvents or posttreatment reagents remain major barriers to their clinical translation.

### Porous and 3D scaffold fabrication

#### Freeze drying technology

Freeze drying is a widely used fabrication technique that employs an ice crystal templating mechanism to construct highly interconnected anisotropic porous structures, offering significant advantages in mimicking the 3D microenvironment of natural cancellous bone [78]. Unlike interfacial polymerization, which primarily modifies material surfaces, or solution casting, which mainly produces film-like constructs, freeze drying regulates the 3D pore network, pore-wall architecture, and spatial distribution of PEDOT-containing phases. Properly controlled freeze drying can generate pore sizes that are favorable for cell infiltration, nutrient diffusion, and vascular ingrowth.

**Fig. 8.**
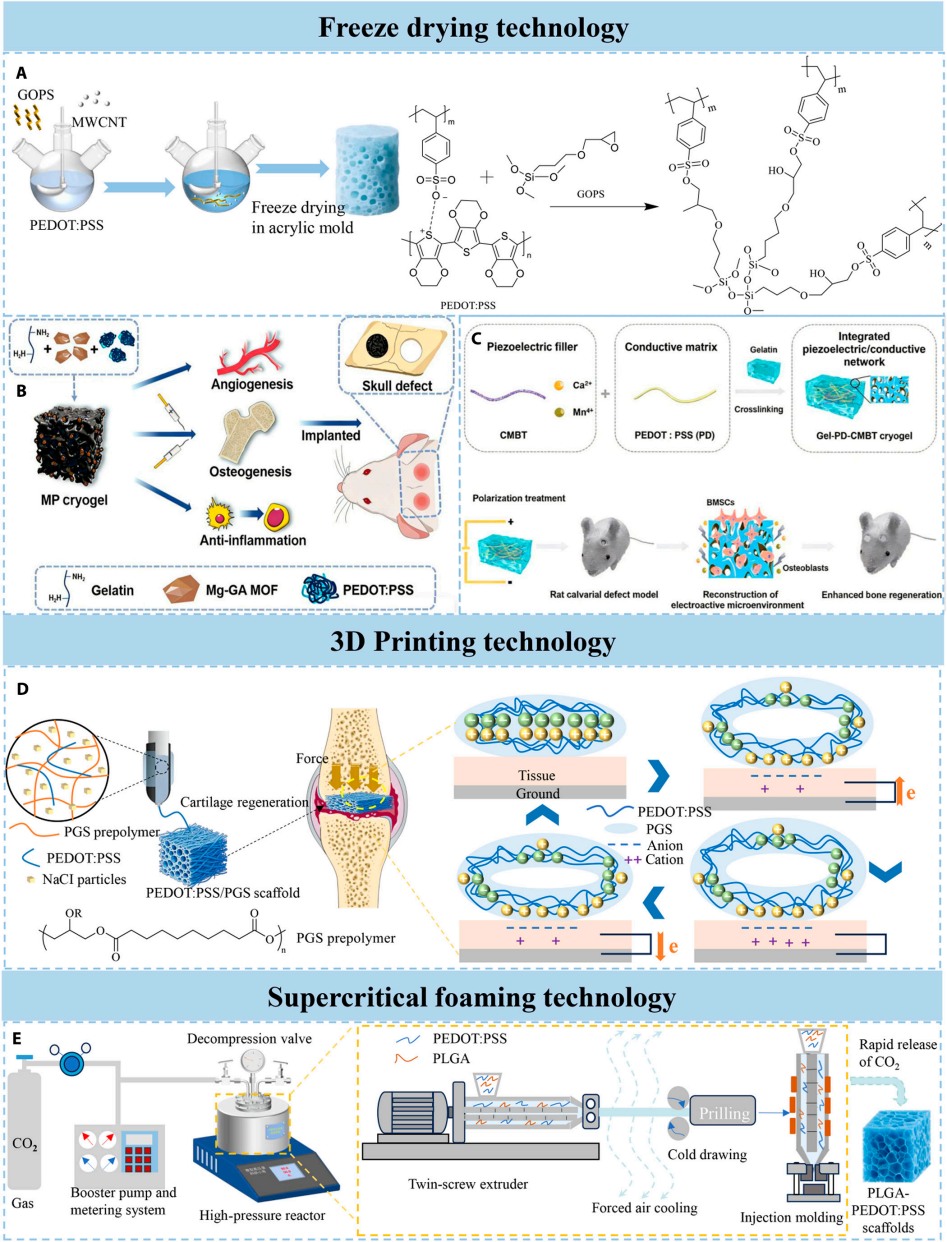
The schematic diagram of (A) poly(3,4-ethylenedioxythiophene):polystyrene sulfonate (PEDOT:PSS)/multiwalled carbon nanotube (MWCNT)/glycidyloxypropyl trimethoxysilane (GOPS) composite scaffold, (B) Mg-gallic acid metal–organic frameworks (Mg-GA MOFs)/PEDOT:PSS cryogel [82] and (C) gelatin (Gel)-PEDOT:PSS-calcium-manganese-doped barium titanate (CMBT) cryogel [83] via freeze drying technology, (D) PEDOT:PSS/poly(glycerol sebacate) (PGS) scaffold via 3D printing technology, and (E) poly(lactic-co-glycolic acid) (PLGA)-PEDOT:PSS scaffold via supercritical foaming technology.

Among the reported freeze-dried PEDOT-based systems, 3 principal design strategies have been adopted. The first strategy focuses on structural stability and osteoconductive porous architecture. For example, the Gel/bioactive glass (BG)/PEDOT scaffold demonstrates that the combination of freeze drying and chemical crosslinking can improve scaffold stability while generating interconnected pores with diameters ranging from 110 to 200 μm, making the scaffold suitable for long-term bone support (over 90 d) [79]. The second strategy emphasizes the construction of conductive pore walls. As illustrated by the PEDOT/MWCNT/GOPS scaffold, conductive components enhance electrical signal transmission throughout the 3D porous network (Fig. 8A) [80]. The third strategy integrates electroactivity with staged biological regulation [81]. Mg-gallic acid metal–organic framework (Mg-GA MOF)/PEDOT freeze gels [82] and Gel/PEDOT codoped barium titanate nanofibers (CMBT) [83] demonstrate that freeze-dried porous networks can simultaneously provide electrical conductivity, ion release, anti-inflammatory regulation, angiogenesis, and piezoelectric charge transfer (Fig. 8B and C). In these systems, PEDOT functions not merely as a conductive additive but as an electroactive component that coordinates structural, chemical, and electrical cues.

However, when PEDOT is unevenly distributed or confined to isolated pore walls, the resulting scaffold may exhibit discontinuous electrical conductivity despite its highly porous architecture. Therefore, freeze-dried PEDOT scaffolds for bone repair should be evaluated not only in terms of total porosity but also with respect to pore interconnectivity, wet-state mechanical retention, and conductivity retention after swelling and degradation. Future developments may combine freeze drying with directional freezing, 3D printing, mineral reinforcement, or post-crosslinking strategies to achieve improved control over pore orientation, mechanical stability, and long-term conductivity retention [84].

#### 3D printing technology

3D printing provides a programmable approach for fabricating PEDOT-based scaffolds with patient-specific geometries, precisely controlled pore architectures, and spatially organized conductive pathways [85]. Compared with solution casting and freeze drying, 3D printing offers superior control over macroscopic geometry, pore size, filament spacing, and gradient organization, making it particularly valuable for the repair of irregular bone defects, osteochondral interfaces, and vascularized porous constructs [86,87]. However, its advantages should not be attributed solely to geometric customization. In PEDOT-containing printable systems, scaffold performance is jointly determined by ink rheology, PEDOT content, postprocessing methods, crosslinking density, printing fidelity, pore interconnectivity, and wet-state conductivity retention.

The central design challenge for 3D-printed PEDOT scaffolds is balancing electrical conductivity with printability. Increasing the PEDOT content promotes conductive network percolation and enhances electrical signal transmission. However, excessive PEDOT loading may increase ink viscosity, impair extrusion stability, cause nozzle clogging, reduce pore interconnectivity, and compromise cytocompatibility.

Representative 3D-printed PEDOT systems illustrate this balance between conductive network formation, scaffold architecture, and biological function. Poly(glycerol sebacate) (PGS)/PEDOT scaffolds demonstrate how programmed microporous architectures and conductive components can be integrated to convert physiological mechanical motion into localized electrical cues (Fig. 8D) [88]. In this system, the hydrophobic microporous structure supports self-powered triboelectric behavior, whereas the PEDOT conductive phase facilitates electrical signal transmission. The scaffold promotes cartilage template formation followed by bone regeneration through an endochondral ossification-related process, highlighting the potential of 3D printing to integrate structural design, functional performance, and time-dependent biological repair. Nevertheless, these systems should be evaluated not only according to their electrical output but also with respect to pore stability, degradation behavior, wet-state conductivity retention, mechanical integrity, and reproducibility under physiological loading.

For bone tissue engineering, 3D printing is particularly suitable for fabricating patient-specific bone scaffolds, osteochondral gradient constructs, vascularized porous architectures, and spatially organized electroactive repair systems [89]. Its principal limitations include the conflict between conductivity and printability, insufficient long-term mechanical strength, complex postprocessing procedures, batch-to-batch variability, and the difficulty of standardizing bioink composition and ES parameters. Therefore, 3D printing should be regarded as a fabrication strategy that integrates scaffold architecture with conductive network design rather than as a universal solution for all PEDOT-based bone repair applications.

#### Supercritical foaming technology

Supercritical foaming provides a relatively mild fabrication strategy with minimal solvent residue for producing porous PEDOT-containing polymer scaffolds [90]. Its primary advantage lies in generating tunable foam-like porous structures while minimizing residual organic solvents, thereby improving implant safety and the biocompatibility of polymer matrices [91]. However, unlike electrochemical polymerization and 3D printing, supercritical foaming has limited capability to generate continuous spatial conductive pathways. During pore nucleation and polymer expansion, PEDOT-rich domains may become isolated or only partially interconnected, resulting in low electrical conductivity and limited electrical output.

The fabrication of poly(lactic-co-glycolic acid)/PEDOT:PSS composite scaffolds using supercritical CO_2_ foaming enables precise control of porosity (up to 50%), pore size (30 to 200 nm), and electrical conductivity (1.0 × 10^−5^ S/cm) through the regulation of processing parameters such as pressure and temperature (Fig. 8E) [92]. This coordinated regulation of processing conditions, scaffold structure, and material performance overcomes several inherent limitations of conventional composite fabrication methods, including pore heterogeneity and performance fluctuations, thereby improving the suitability of these materials for the regeneration of electrically responsive tissues that require specific structural and conductive properties, such as neural and cardiac tissues. Future studies should combine supercritical foaming with postfoaming PEDOT coating, mineral reinforcement, or directional pore control to overcome the inherent trade-off between porosity and electrical conductivity.

### Cross-method comparison and route selection

The fabrication strategies for PEDOT-based systems can be classified into 3 translational categories. Interfacial methods, including electrochemical polymerization and VPP, are most suitable for applications targeting osseointegration and implant surface functionalization because they produce compact conductive coatings with low interfacial impedance. However, these methods contribute minimally to bulk defect filling and remain limited by substrate conductivity, coating thickness, and depth-dependent diffusion. Matrix-forming methods, including solution casting and crosslinking, are more appropriate for soft, hydrated repair systems because they facilitate the incorporation of natural polymers, inorganic ions, and therapeutic molecules. Their primary limitation is that the PEDOT-rich conductive phase may be diluted or immobilized within insulating matrices, resulting in a trade-off between electrical conductivity and mechanical performance, particularly in hydrogel systems.

Architecture-forming methods, including electrospinning, 3D printing, freeze drying, and supercritical foaming, are more suitable for reconstructing tissue-scale architectures. Electrospinning provides ECM-like nanofibrous cues that promote cell alignment but generally requires additional conductive coatings to achieve through-thickness electrical signal transmission. 3D printing enables the fabrication of patient-specific geometries and the programmable distribution of conductive networks. However, increasing the conductive component often compromises printability and the viability of cell-laden bioinks. Freeze drying efficiently generates porous architectures that facilitate cell infiltration and vascular ingrowth, although the resulting high porosity may disrupt continuous conductive pathways and reduce load-bearing capacity. Supercritical foaming minimizes solvent residues and provides a mild fabrication process, whereas currently available PEDOT-containing foams generally exhibit limited electrical conductivity because the conductive domains remain isolated. Therefore, the fabrication strategy should be selected according to the intended application, such as conductive coatings for interfacial integration, soft hydrogels for conformal repair, fibrous scaffolds for contact guidance, 3D-printed constructs for patient-specific defects, and porous cryogels or foams for cell infiltration and staged release.

## Applications of PEDOTs in Bone Tissue Engineering

### Osteogenic repair

Bone defect repair remains a major challenge because successful osteogenesis requires the coordinated regulation of cell adhesion, osteogenic differentiation, vascular invasion, mineral deposition, and mechanical remodeling [93]. PEDOT-based scaffolds contribute to osteogenic repair not merely by enhancing electrical conductivity but by constructing electroactive interfaces that integrate electrical cue transmission, scaffold architecture, and the controlled release of bioactive components. Their osteogenic effects are achieved through 3 interconnected mechanisms: mixed ionic and electronic signal transmission, structure-mediated cell guidance, and filler- or ion-mediated biological regulation.

**Fig. 9.**
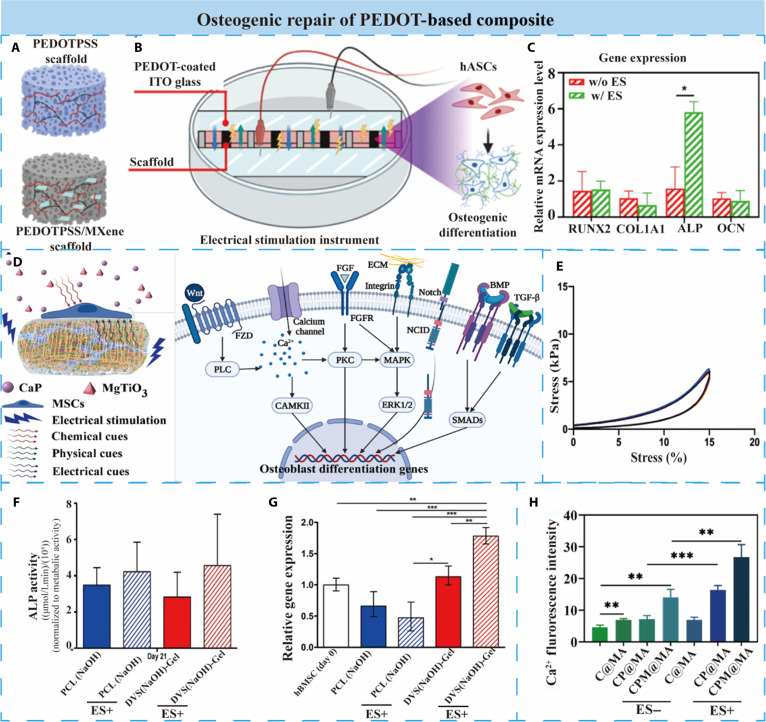
Microstructure schematic diagram of (A) poly(3,4-ethylenedioxythiophene):polystyrene sulfonate (PEDOT:PSS) and PEDOT:PSS/MXene scaffolds, (B) the electrical stimulation (ES) culture platform, (C) Expression of osteogenic-specific gene markers in control and ES groups after 14-d differentiation culture [69]. (D) The osteogenic differentiation mechanism scheme of calcium phosphate PEDOT:PSS-magnesium titanate-methacrylated alginate (CPM@MA) hydrogels under ES [35]. (E) The loading and unloading curves of the PEDOT:PSS/multiwalled carbon nanotube (1 wt.%)/glycidyloxypropyl trimethoxysilane (GOPS) scaffold under a 15% strain and 100 cycles [80]. The changes in (F) cellular metabolic activity and (G) the expression of the calcium voltage-gated channel subunit alpha1 C (CACNA1C) after 21 d under the quantitative measurement of calcium content [100]. (H) Quantitative fluorescence intensity of hMSCs in various CPM@MA hydrogel groups with/without ES [35].

Among porous PEDOT-based scaffolds, osteogenic enhancement is generally determined not by electrical conductivity alone but by the synergistic interaction among conductive pathways, pore architecture, and the release of bioactive fillers [94]. In addition, functional ions released from conductive materials gradually activate osteogenesis-related signaling pathways. For example, PEDOT:PSS/MXene composite sponges fabricated by freeze templating form interconnected pores that mimic the trabecular bone architecture, thereby promoting the adhesion and migration of human adipose-derived stem cells (hASCs) (Fig. 9A) [69]. Furthermore, the 2-dimensional sheet-like structure of MXene enhances focal adhesion formation, whereas its surface gradually releases titanium ions (Ti^3+^/Ti^4+^), thereby activating the transforming growth factor-β/BMP signaling pathway and up-regulating ALP expression (Fig. 9B and C). Similarly, the porous architecture of CPM@MA hydrogels provides a 3D growth environment for BMSCs, with an ultimate load capacity of 35.6 MPa, corresponding to 83.3% of that of native bone [35]. Mg^2+^ released from the hydrogel inhibit matrix metalloproteinases, reduce cartilage degradation, and deliver mild ES that suppresses chondrocyte apoptosis (Fig. 9D). However, the incorporation of MXene nanosheets may partially interrupt or hinder the formation of continuous conductive networks among PEDOT:PSS chains, thereby reducing the overall electrical conductivity of the scaffold. Therefore, optimizing the dispersion of MXene represents an important direction for future studies to achieve an appropriate balance among electrical conductivity, mechanical performance, and porosity.

PEDOT:PSS systems containing MWCNTs further demonstrate the existence of a balance between electrical conductivity and cytocompatibility [95]. In the PEDOT:PSS/MWCNT/GOPS system, the addition of 3 wt.% MWCNTs significantly increased the electrical conductivity to 14 S/cm [80]. This improvement is attributed to the ability of 1-dimensional MWCNTs to form continuous conductive pathways within the PEDOT:PSS matrix, thereby bridging insulating regions. However, excessive MWCNT loading may induce cytotoxic effects, reducing cell viability to approximately 70%. By contrast, the PEDOT:PSS/MWCNT (1 wt.%)/GOPS scaffold achieved the optimal balance between electrical conductivity (1.0 S/cm) and cell viability (>90%). In this scaffold, water stability increased by 30%, whereas the swelling ratio decreased from 2,900% to 2,300%. Moreover, the scaffold maintained its structural integrity after 100 compression cycles at 15% strain, demonstrating clinically relevant durability for long-term implantation (Fig. 9E). The porous architecture of the PEDOT:PSS/MWCNT/GOPS scaffold facilitates cell infiltration, dexamethasone (DEX) loaded into the MWCNTs enables sustained drug release and prolonged osteogenic signaling, and the osteogenic enhancement induced by ES acts synergistically to overcome the limitations of conventional bone repair scaffolds, which primarily provide structural support without actively regulating cellular differentiation.

Similarly, composites composed of Sr and Mg codoped HA with PEDOT:PSS form bilayer interconnected porous coatings [96]. This architecture facilitates continuous nutrient transport and metabolic waste removal, thereby providing osteoblasts with a 3D microenvironment that enhances intercellular communication and accelerates bone matrix deposition. Mechanistically, Sr^2+^ ions suppress osteoclast activity while stimulating osteoblast proliferation, whereas Mg^2+^ ions regulate ALP activity. Their combined effects synergistically promote mineralized nodule formation through activation of the phosphatidylinositol 3-kinase/protein kinase B signaling pathway and up-regulation of collagen type I alpha 1 chain expression. The excellent electrical conductivity of PEDOT, together with the superior biocompatibility of Sr- and Mg-doped HA, provides synergistic effects in corrosion resistance and osseointegration. Additional bioactive elements, including Ag for antibacterial activity, Zn for angiogenesis promotion, and Si for vascular growth, can also be incorporated into the coating to confer multiple biological functions, including antibacterial and anti-infective activities.

Furthermore, PEDOT-rich conductive domains can transmit externally applied or self-generated electrical cues to bone-related cells, thereby regulating membrane potential and Ca^2+^-related signaling [97]. This process is closely associated with the up-regulation of osteogenic markers, including ALP, RUNX2, OCN, BMP/Smad, and COL I, as well as enhanced ECM mineralization [98,99]. For example, Silva et al. [100] treated the surface of NaOH-pretreated PCL scaffolds with PEDOT:PSS, GOPS, Gel, and divinyl sulfone (DVS) to fabricate bioactive scaffolds. ALP activity was slightly increased in the ES group. Calcium quantification demonstrated that calcium deposition in the DVS (NaOH)-Gel/ES group was 2.29-fold higher than that in the corresponding group without ES and 2.46-fold higher than that in the PCL (NaOH)/ES group, indicating a significant synergistic effect (Fig. 9F). In the DVS (NaOH)-Gel/ES group, the osteogenesis-related genes COL I, OCN, and calcium voltage-gated channel subunit alpha1 C (CACNA1C) were significantly up-regulated (Fig. 9G). Notably, the up-regulation of CACNA1C suggests that ES may promote osteogenesis through activation of the calcium signaling pathway. Similarly, when combined with ES (200 mV/cm, 0.1 Hz), PEDOT:PSS/MXene scaffolds increased intracellular Ca^2+^ levels in hASCs by 5.79-fold and enhanced ALP activity to 2.2-fold that of the nonstimulated control group (Fig. 9H) [35]. However, the mechanical strength of the PEDOT:PSS/MXene scaffold, with a maximum compressive modulus of approximately 100 kPa, may be insufficient for the repair of load-bearing bone defects. Therefore, future studies may need to enhance the mechanical performance of these scaffolds through structural optimization, such as the incorporation of dense reinforcing layers or additional strengthening materials.

In addition, the dual-regulation strategy that combines material-induced osteogenesis with physical stimulation has been widely adopted in 3D conductive scaffolds [101]. For example, PEDOT:PSS/MXene scaffolds exhibit the dual advantages of electroactivity and osteoinductive capacity [69]. hASCs cultured on these scaffolds showed significantly higher ALP, a mid-stage osteogenic marker, activity after 14 d than nonstimulated controls, together with enhanced immunofluorescence signals of RUNX2, an early osteogenic marker. These findings collectively indicate that ES accelerates the transition of hASCs from osteoprogenitor cells to functional osteoblasts. In another study, the Gel/BG/PEDOT scaffold exhibited a significantly greater proportion of new bone formation than the Gel/BG scaffold, effectively promoting bone regeneration [102]. The incorporation of PEDOT:PSS modulates the local electrical microenvironment, thereby activating osteogenesis-related signaling pathways. This process promotes new bone growth from the defect margins toward the center, providing both structural support and regenerative capacity for bone defect repair. Concurrently, BG releases bioactive ions that further induce osteogenic differentiation.

In summary, these studies demonstrate that the osteogenic performance of PEDOT-based systems does not follow a simple linear relationship in which higher electrical conductivity results in superior bone regeneration. Instead, osteogenic efficacy depends on whether the conductive network remains sufficiently continuous while simultaneously maintaining pore accessibility, cytocompatibility, ion transport, and mechanical stability. Existing strategies mainly rely on physical doping, such as the incorporation of MXene nanosheets or carbon nanotubes. Although these approaches locally enhance electrical signal transmission and provide ion-release functions, such as Mg^2+^-mediated activation of osteogenic pathways, the introduction of rigid 2-dimensional or 1-dimensional nanomaterials often disrupts the continuity of PEDOT:PSS chains, resulting in a marked reduction in macroscopic electrical conductivity and mechanical anisotropy. To overcome this mechanoelectrical coupling bottleneck, future composite network designs should shift from physical blending toward molecular-level orthogonal crosslinking and self-driven piezoelectric coupling [103]. For example, a photocrosslinked backbone based on rigid natural macromolecules, such as lignin, can be constructed using photoinitiators to provide compressive fatigue resistance comparable to that of the cartilage and subchondral bone layers. Simultaneously, a flexible PEDOT:PSS conductive network can be formed in situ within this framework. More importantly, by precisely anchoring piezoelectric crystals, such as piezoelectric small molecules with chondroinductive activity, within this dual-network system, a passive electroactive microenvironment can be established. Under the cyclic mechanical loading associated with normal joint activity, transient local electrical potentials generated by the piezoelectric crystals can be efficiently transmitted through the continuous PEDOT network to deeper tissues, thereby enabling the 3D synergistic delivery of mechanical, electrical, and biochemical signals without compromising the macroscopic mechanical strength.

### Osteochondral interface and subchondral bone repair

Although PEDOT-based systems have also been investigated for cartilage repair, cartilage-related studies are discussed here only from a bone-centered perspective. In osteochondral defects, successful regeneration requires not only restoration of the cartilage layer but also reconstruction of the calcified cartilage zone, subchondral bone, and the electromechanical coupling across the osteochondral interface [104]. Therefore, this section focuses on the roles of PEDOT-based conductive scaffolds in osteochondral interface repair, subchondral bone remodeling, and coordinated chondrogenic and osteogenic regulation rather than providing a comprehensive overview of cartilage tissue engineering.

In osteochondral repair, the role of PEDOT extends beyond providing external ES. Instead, PEDOT can function as an artificial charge-transfer pathway that compensates for the disrupted electromechanical microenvironment across the cartilage and subchondral bone interface [17]. As a representative strategy for cartilage-layer reconstruction, a poly(4-(2,3-dihydrothieno[3,4-b][1,4]dioxin-2-ylmethoxy)butane-1-sulfonic acid sodium salt (PEDOT-S)/collagen composite scaffold (PEDOT-ACS) was developed [105]. The PEDOT-ACS scaffold exhibits a hydrated compressive modulus of 0.529 MPa, comparable to that of native cartilage, while its electrical conductivity enables the delivery of mild electrical cues that reduce chondrocyte apoptosis. The enhanced negative surface charge of PEDOT-ACS is likely to mediate electrostatic interactions that influence the binding of cell adhesion molecules to the scaffold, thereby regulating cell adhesion, spreading, and morphology to create a favorable microenvironment for improved cell viability and differentiation. With a zeta potential of −19.9 mV, PEDOT-ACS can further regulate cell adhesion, spreading, and glycosaminoglycan production through its surface charge and electroactivity. However, this study did not directly measure the bulk electrical conductivity of the composite scaffold or apply exogenous ES, making it impossible to determine whether the electrical activity directly contributed to the observed chondrogenic effects. Furthermore, the distribution of PEDOT-ACS within the collagen matrix may be heterogeneous, potentially limiting the overall electrical conductivity. Moreover, because this study primarily focused on chondrocyte behavior and cartilage matrix production, its relevance to bone-centered osteochondral repair should be regarded as evidence supporting cartilage-layer restoration within the osteochondral unit rather than as direct evidence of subchondral bone regeneration.

**Fig. 10.**
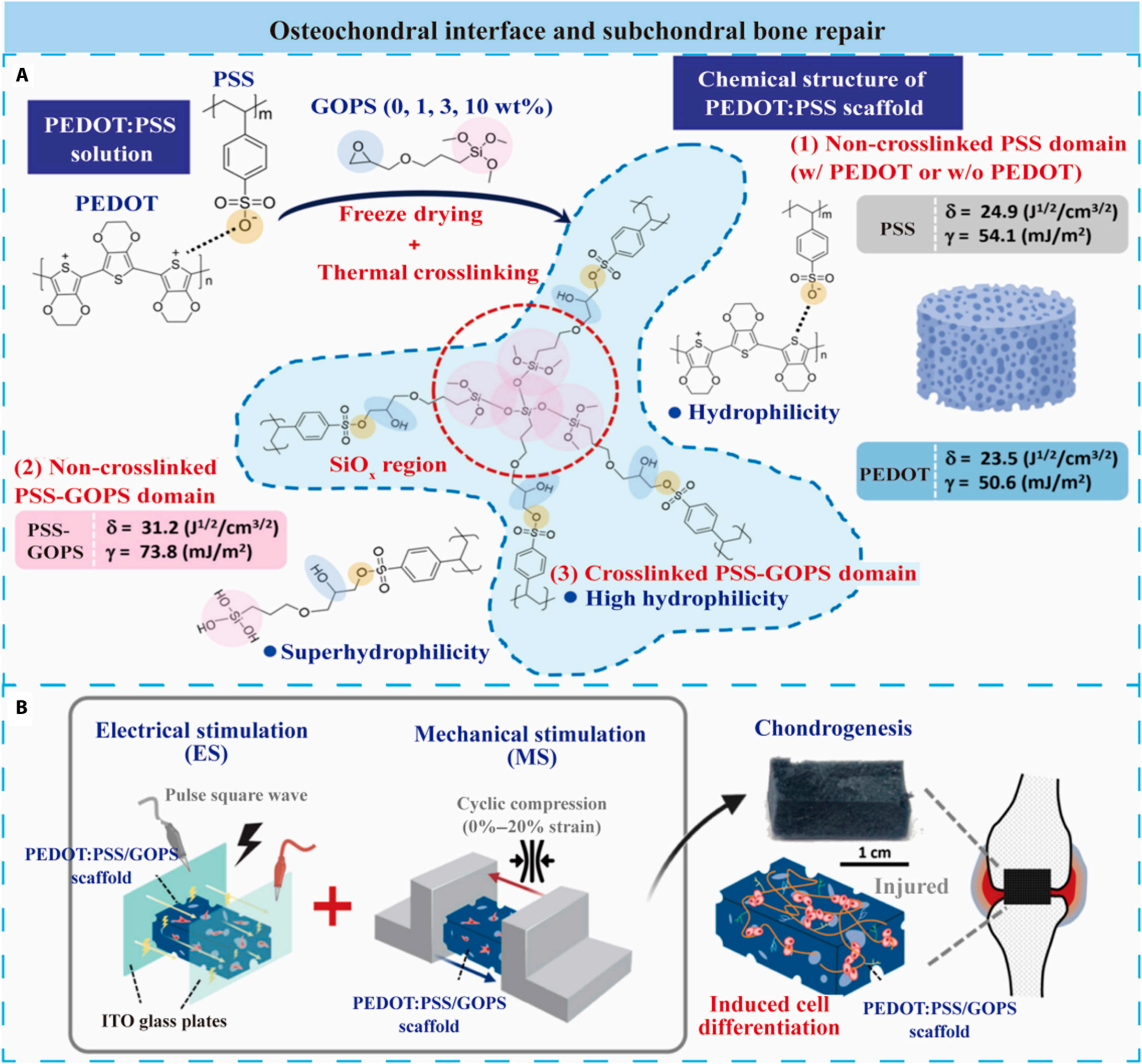
Schematic diagram of the synthesis of (A) poly(3,4-ethylenedioxythiophene):polystyrene sulfonate (PEDOT:PSS)/glycidyloxypropyl trimethoxysilane (GOPS) scaffolds and (B) the osteogenic mechanism under electrical stimulation (ES) and mechanical stimulation [106].

Dynamic mechanical and electrical cues are also critical for osteochondral interface repair because the osteochondral unit is continuously subjected to cyclic loading during normal daily activities. Liu et al. [106] fabricated a PEDOT:PSS/GOPS scaffold using the freeze drying method (Fig. 10A). This scaffold exhibits several favorable characteristics, including superhydrophilicity, high porosity (70.5%), good mechanical stability with a Young’s modulus of 11.5 kPa, suitable electrical conductivity (5.6 × 10^−6^ S/cm), and low cytotoxicity. Furthermore, the scaffold promotes chondrocyte differentiation through dual-responsive stimulation consisting of mechanical stimulation (1 Hz, 20% strain) and ES (5 V/cm, 5 Hz, 8-ms pulse width) (Fig. 10B). Among these stimuli, mechanical stimulation, which mimics physiological loading conditions in vivo, induced a more pronounced up-regulation of the chondrogenic marker gene SRY-box transcription factor 9 (SOX9), with a 3.1-fold increase. In contrast, ES exhibited a greater capacity to enhance collagen type II alpha 1 chain expression, resulting in a 3.6-fold increase. These findings indicate that mechanical and electrical cues regulate distinct aspects of cartilage matrix formation. From the perspective of osteochondral repair, this system is relevant because the maintenance of a functional cartilage-like matrix is essential for restoring the upper layer of the osteochondral interface. Nevertheless, further evidence regarding calcified cartilage formation, subchondral bone remodeling, and long-term osteochondral interface integration is still required.

In the reported studies on PEDOT-based systems, the PGS/PEDOT:PSS scaffold provides a more direct bone-centered strategy, in which cartilage formation serves as an intermediate template rather than the final repair endpoint [107]. To address the severe restriction of cell survival and function caused by the hypoxic and avascular microenvironment of bone defects, Ma et al. fabricated a PGS/PEDOT:PSS composite scaffold with a hierarchical porous architecture using 3D printing technology. This scaffold harvests mechanical energy generated during physiological activities and produces a triboelectric effect through the contact and separation between the PGS matrix and PEDOT:PSS, thereby generating ES of approximately 5 V without the need for an external power source. BMSCs were induced toward chondrogenic differentiation on the triboelectric scaffold for up to 4 weeks, successfully forming tissue-engineered cartilage expressing characteristic cartilage markers, including collagen type II and SOX9. By employing cartilage as an intermediate template for bone regeneration and adopting an endochondral ossification strategy, the scaffold achieved significant repair of bone defects.

Collectively, these studies suggest that PEDOT-based osteochondral scaffolds should not be evaluated solely according to chondrogenic markers or electrical conductivity [108]. Their translational potential depends on their ability to coordinate cartilage-layer restoration, calcified cartilage formation, subchondral bone remodeling, and stable electromechanical coupling under cyclic loading. One of the major challenges is balancing cartilage-like hydration and viscoelasticity with the continuity of the conductive network. Excessive hydrogel dilution may improve cartilage-like mechanical properties but disrupt PEDOT-rich conductive pathways, whereas excessive PEDOT content may compromise biomimetic compliance, cell infiltration, and ECM secretion. In addition, the limited degradability of CPs and the potential release of nondegradable PEDOT-rich fragments during long-term joint loading raise safety concerns in the confined joint environment. Therefore, future PEDOT-based osteochondral systems should integrate controlled electrical conductivity, gradient mechanical support, stable pore and interfacial architectures, and well-defined degradation or clearance pathways to bridge the translational gap between cartilage-layer repair and subchondral bone regeneration.

### PEDOT-enabled osteoporotic bone repair and orthopedic sensing

In degenerative orthopedic diseases, PEDOT-based systems have expanded from general bone defect repair to regenerative therapy and functional monitoring for specific orthopedic disorders [109–111]. From the perspective of orthopedic applications, 2 major directions are particularly important. The first is osteoporotic bone repair, in which conductive scaffolds are designed to restore the osteogenic capacity of endogenous stem cells [112]. The second is orthopedic sensing, in which stretchable PEDOT-based hydrogels are used to monitor joint movement and disease progression. These 2 applications illustrate the distinct functions of PEDOT. In bone repair, PEDOT serves as a regenerative electroactive interface, whereas in orthopedic sensing, it functions as a soft bioelectronic network for physiological monitoring.

**Fig. 11.**
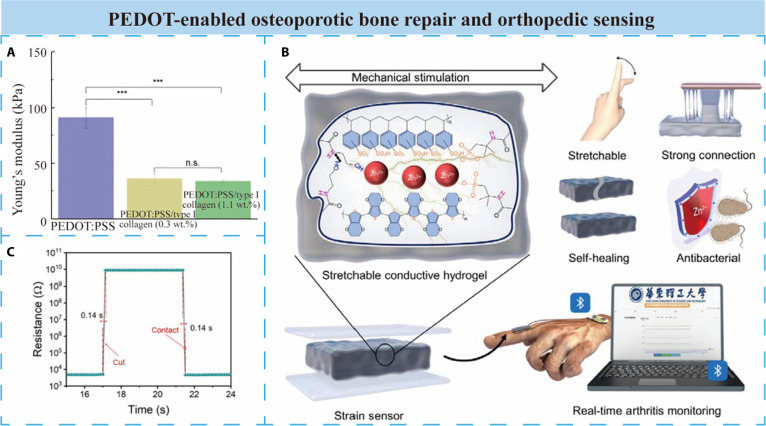
(A) Elastic modulus of poly(3,4-ethylenedioxythiophene):polystyrene sulfonate (PEDOT:PSS) and PEDOT:PSS/type I collagen (COL I) (0.3 wt.%, 1.1 wt.%) scaffold [113]. (B) Schematic diagram of the synthesis of PHAC hydrogel. (C) The cutting–healing cycle process of the conductive properties at the same location of the PHAC hydrogel [115]. n.s., not significant.

For osteoporotic bone repair, the primary challenge is to enhance bone formation while compensating for the impaired osteogenic potential of endogenous stem cells. A representative strategy involves the fabrication of porous PEDOT/COL I scaffolds by freeze drying, in which COL I provides a bone matrix-mimicking microenvironment while PEDOT introduces electroactivity [113]. This scaffold exhibits a Young’s modulus of 33.5 kPa while maintaining electroactivity (Fig. 11A). Notably, neural crest-derived stem cells cultured on the PEDOT:PSS/COL I scaffold exhibited robust proliferation and underwent osteogenic differentiation without requiring preconditioning. In particular, the scaffold containing 0.3 wt.% collagen exploited the synergistic effects of biomimetic mechanical cues and ES, resulting in a significant increase in mineralized matrix deposition. However, collagen may aggregate in the acidic PEDOT:PSS dispersion, leading to heterogeneous distribution within the scaffold and consequently affecting the uniformity of cellular responses. Therefore, future studies should optimize PEDOT-collagen mixing, wet-state conductivity retention, mineral reinforcement, and the balance between biomimetic compliance and mechanical support.

In contrast, PEDOT-based orthopedic sensors are primarily intended for disease monitoring rather than direct tissue regeneration. Rheumatoid arthritis is characterized by joint stiffness and reduced range of motion, making real-time motion monitoring valuable for disease assessment and rehabilitation evaluation [114]. The poly(N-hydroxyethyl acrylamide-co-2-acrylamido-2-methyl-1-propanesulfonic acid)/PEDOT/Zn^2+^ conductive hydrogel (PHAC) sensor represents a typical example of this diagnostic strategy (Fig. 11B) [115]. The PHAC sensor exhibits exceptional stretchability (5,200%), strong adhesion (61.4 kPa on Cu), rapid self-healing capability, restoring electrical conductivity within 0.14 s (Fig. 11C), and antibacterial activity. Integrated with a custom-designed printed circuit board and a Bluetooth module, the sensor enables real-time monitoring of joint movements, such as those of the fingers and wrists. It can detect subtle changes in range of motion associated with the early morning stiffness characteristic of rheumatoid arthritis, thereby providing patients with a lightweight and wearable tool for daily disease monitoring. Nevertheless, its translational potential depends less on short-term stretchability than on long-term signal stability under conditions involving perspiration, temperature fluctuations, repeated mechanical deformation, and environments resembling synovial fluid.

Taken together, PEDOT-based strategies for degenerative orthopedic diseases involve 2 distinct but closely related translational trade-offs [116]. For osteoporotic bone repair, electroactive scaffolds must achieve an appropriate balance among electrical conductivity, osteogenic stimulation, mineral reinforcement, and load-bearing stability. Although soft conductive matrices effectively activate stem cell responses, they often fail to provide sufficient immediate mechanical support for fragile trabecular bone. In orthopedic sensing, highly stretchable and self-healing PEDOT hydrogels must balance deformability with low hysteresis, stable baseline electrical signals, swelling resistance, and reproducible calibration during long-term operation. Therefore, future PEDOT-based systems for degenerative orthopedic diseases should be designed according to their intended applications, either as regenerative bone scaffolds requiring stable mechanical and electrical performance or as wearable or implantable sensors requiring durable signal fidelity and excellent biocompatibility.

### Integrated bioelectronic regulation for bone regeneration

Bone regeneration is a temporally coordinated process involving the sequential stages of early inflammatory regulation, vascular invasion, osteogenic differentiation, matrix mineralization, and tissue remodeling [117]. Therefore, multifunctional PEDOT-based scaffolds should not be regarded simply as combinations of conductive, angiogenic, antibacterial, and osteogenic components [118]. Instead, these functions should be spatially and temporally coordinated with the natural bone healing cascade. In this context, the value of PEDOT extends beyond its electrical conductivity to its ability to function as a redox-active interface with mixed ionic and electronic conductivity that integrates ES, cell guidance, drug release, ion delivery, and microenvironmental regulation [119,120].

**Fig. 12.**
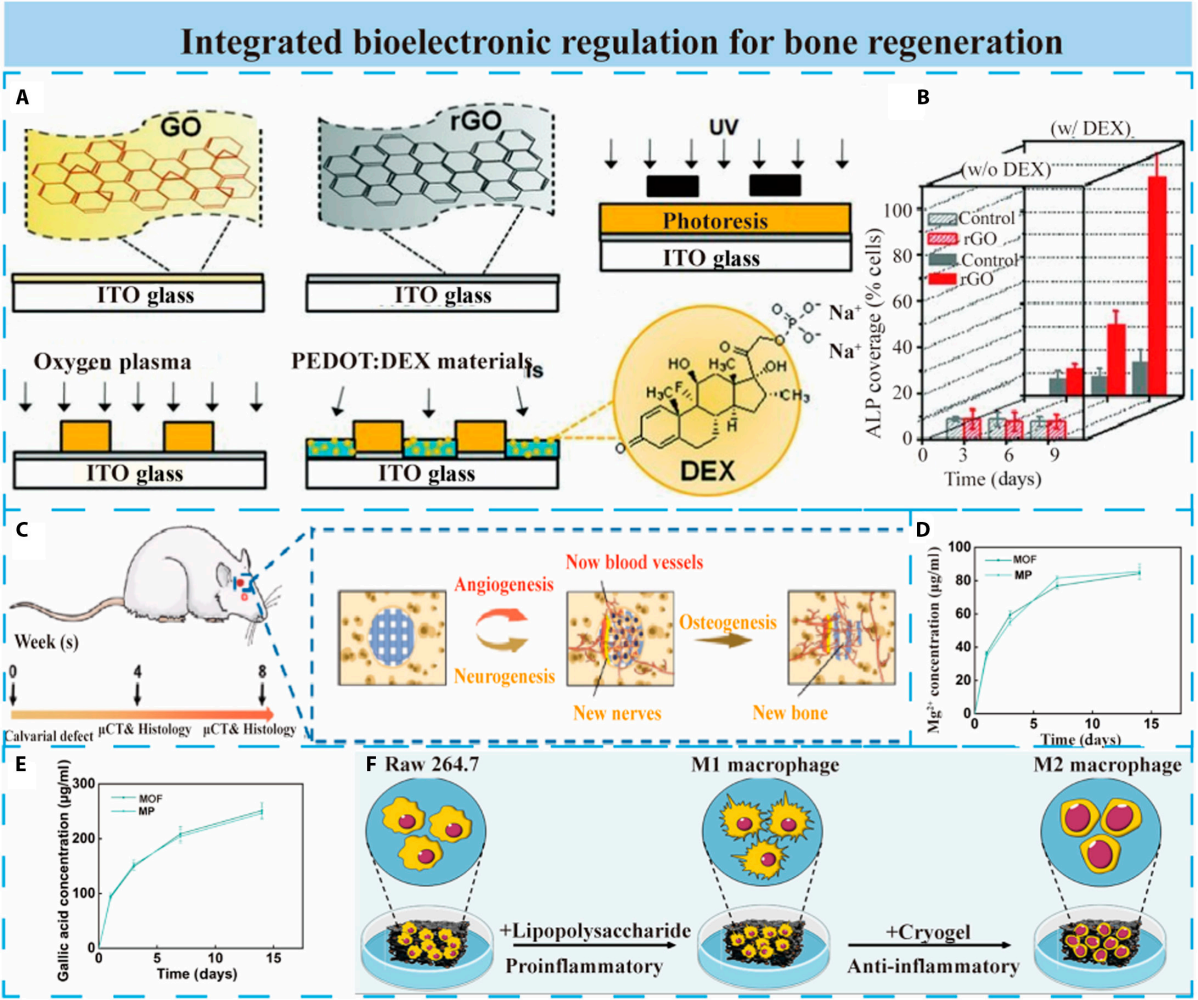
(A) Schematic diagram of the reduced graphene oxide (rGO)-PEDOT device loaded with dexamethasone (DEX). (B) The alkaline phosphatase (ALP) expression of differentiated hMSCs on different substrates was quantified by alizarin red staining [47]. (C) The schematic diagram illustrating the promotion of nerve growth and bone regeneration in rats after Gel:HA:HAp scaffold implantation [123]. The release amounts of (D) Mg^2+^ and (E) GA in metal–organic framework (MOF) and MP cryogel. (F) A schematic diagram illustrating the process by which RAW264.7 cells promote the transformation of M1 macrophages into M2 macrophages [82]. UV, ultraviolet.

Current evidence does not support a universal numerical conductivity threshold for PEDOT-based bone scaffolds because conductivity values are measured under different hydration states, frequencies, scaffold geometries, and biological models. Nevertheless, the reviewed studies suggest the existence of a route-specific functional conductivity window. Below the percolation threshold, discontinuous PEDOT domains cannot efficiently transmit electrical cues. Once a continuous mixed ionic/electronic network is established, further increases in PEDOT content may provide diminishing biological benefits while compromising cell compatibility, pore accessibility, hydrogel hydration, ion transport, printability, or degradation behavior. Therefore, PEDOT-based bone repair systems should not be optimized solely by maximizing conductivity. Instead, conductivity should be balanced with scaffold architecture, mechanical compliance, degradation kinetics, and the specific biological functions required at each stage of bone regeneration. Electroresponsive release represents one of the most distinctive functions of PEDOT-based systems. By combining PEDOT with rGO, conductive microelectrode arrays can integrate topographical guidance with electrically controlled drug delivery. In the rGO-PEDOT microelectrode system, rGO acts as an adhesion and alignment layer, guiding hMSC orientation, whereas PEDOT serves as a redox-active drug-release layer for DEX loading and release (Fig. 12A). Under cyclic electrical stimulation from −1 to +1 V, DEX release was triggered in a time-dependent manner, and osteogenic ALP expression increased by approximately 3- to 5-fold within 9 d (Fig. 12B) [47]. This example illustrates how PEDOT can coordinate cell orientation and osteogenic induction through an electroresponsive interface. However, the evidence was obtained on indium tin oxide-based planar electrodes rather than biodegradable 3-dimensional implants. Therefore, its translational relevance depends on whether similar electrically triggered release can be achieved in degradable, porous, and mechanically stable bone scaffolds.

Multifunctional integration can also be used to couple osteogenesis with angiogenesis and neurovascularization [118]. Piezoelectric and ion-releasing scaffolds provide a representative route [121]. For example, piezoelectric whitlockite/PCL scaffolds combine piezoelectric whitlockite with a printable PCL framework, allowing the scaffold to partially mimic the electromechanical microenvironment of natural bone [122]. The piezoelectric phase can generate bone-like electrical cues under mechanical loading, while Mg^2+^ and Ca^2+^ release contributes to angiogenic and osteogenic regulation. In this system, Runx2 expression increased by approximately 4 times, vascular endothelial growth factor expression increased by approximately 3 times, and a rat calvarial defect model showed enhanced bone formation and neurovascularization after 8 weeks. Similarly, PEDOT-NPs embedded in a Gel/HA/hydroxyapatite (HAp) matrix can form a porous conductive scaffold with interconnected pores, in which PEDOT-NPs support electrical signal transmission and the matrix provides a biomimetic environment for hBMSC proliferation, osteogenic differentiation, and provascular factor secretion (Fig. 12C) [123]. These examples suggest that PEDOT-based multifunctional scaffolds are most effective when electrical cues are coordinated with pore architecture, ion release, and vascular support.

Immunomodulatory integration is another critical direction because excessive early inflammation can suppress vascular invasion and osteogenic differentiation [124,125]. Mg-GA/MOF/Gel/PEDOT cryogels provide a representative example of inflammation–angiogenesis–osteogenesis coupling [82]. In this system, Mg-GA MOFs enable sustained release of Mg^2+^ and GA, with cumulative release reaching approximately 80 μg/ml Mg^2+^ and 240 μg/ml GA (Fig. 12D and E). GA promoted macrophage polarization from the M1 to M2 phenotype, reducing interleukin-6 expression by 67.1% and increasing Arg-1 expression by 13.5-fold (Fig. 12F). Meanwhile, Mg^2+^ enhanced angiogenesis formation by 2.3-fold and increased ALP activity by 2-fold. Together with the conductivity of PEDOT (1.21 × 10^−3^ S/cm) and the porous cryogel structure (100 to 200 μm), the scaffold provided an electroactive and immunoregulatory microenvironment for bone repair. In a rat calvarial defect model, this multifunctional scaffold achieved higher bone volume/total volume and bone mineral density after 8 weeks than single-function controls. This research highlights that PEDOT-based multifunctional systems should be evaluated by coordinated biological timing rather than by the presence of multiple functions alone.

In these studies, it can be observed that the multifunctional PEDOT-based scaffolds should shift from static component stacking to spatiotemporal coordination. The major challenge is that different functions may have conflicting material requirements. High loading of MOFs, BG, piezoelectric particles, or other inorganic fillers may improve ion release or electromechanical activity, but it can also interrupt PEDOT-rich conductive pathways, induce filler aggregation, reduce pore accessibility, or weaken mechanical integrity after degradation. Similarly, anti-inflammatory, angiogenic, and osteogenic factors should not necessarily be released simultaneously. Bone healing requires an ordered sequence of moderate inflammation, vascular ingrowth, osteogenic differentiation, and mineralization. Burst release or mismatched release kinetics may disturb this sequence and reduce regenerative efficiency.

Therefore, future multifunctional PEDOT-based bone scaffolds should be designed according to a spatiotemporal orthogonality principle [126]. Anti-inflammatory factors should primarily regulate the early inflammatory phase, angiogenic cues should support vascular invasion, and osteogenic ions or molecules should be sustained during matrix formation and mineralization. Electrically triggered release should also consider possible Faradaic reactions, local pH changes, and growth-factor stability under ES. To bridge the translational gap, future studies should report degradation half-life, mass loss, conductivity retention, electrical-output retention, release half-times, and stage-specific biological outcomes together, rather than evaluating conductivity, biodegradation, and biological efficacy as independent endpoints.

### Translational design rules for PEDOT-based bone repair systems

Based on the PEDOT-based systems summarized above and in Table 2, the biological performance of PEDOT scaffolds should be interpreted through specific design rules rather than isolated material examples. Across the studies, in vitro and in vivo outcomes of PEDOT-based bone repair systems appear to be governed by different dominant factors. In vitro, conductive coatings, hydrogels, fibrous scaffolds, and porous constructs frequently enhance cell adhesion, proliferation, Ca^2+^-related signaling, osteogenic gene expression, and mineralized matrix deposition once a conductive interface or continuous PEDOT-rich domain is formed. However, these favorable cellular responses do not necessarily translate proportionally into in vivo bone regeneration. In vivo performance is additionally controlled by coating adhesion, pore interconnectivity, vascular invasion, immune response, degradation kinetics, mechanical retention, and conductivity stability under physiological loading. Therefore, different PEDOT scaffold classes should be evaluated according to route-specific translational criteria. The interfacial coatings require long-term adhesion and charge-transfer stability. Hydrogels require swelling/degradation-coupled conductivity retention. Fibrous scaffolds require through-thickness signal transmission and compression resistance. In addition, porous or 3D-printed constructs require pore-wall conductive continuity, vascular integration, and mechanical durability.

Current evidence does not support a universal numerical conductivity threshold for PEDOT-based bone scaffolds, because conductivity values are measured under different hydration states, frequencies, scaffold geometries, and biological models. Nevertheless, the reviewed studies suggest the existence of a route-specific functional conductivity window. Below the percolation threshold, discontinuous PEDOT domains cannot efficiently transmit electrical cues. After a continuous mixed ionic/electronic network is formed, further increases in PEDOT content may yield diminishing biological returns and may compromise cell compatibility, pore accessibility, hydrogel hydration, ion transport, printability, or degradation behavior. Thus, the PEDOT-based bone repair system should not be optimized based on the principle that the higher the conductivity, the better. Instead, the conductivity should be balanced with the scaffold structure, mechanical compliance, degradation kinetics, and the specific biological functions at each stage.

## Conclusion

PEDOTs have emerged as a distinctive class of electroactive polymer platforms for bone tissue engineering because they combine mixed electronic/ionic conductivity, excellent processability, mechanical compliance, and compatibility with composite scaffold design. The value of PEDOT-based systems extends beyond serving as conductive fillers. Rather, they function as bioelectronic interfaces that integrate fabrication-derived structures, local electrical signaling, cell–material interactions, and staged tissue regeneration.

The principal conclusion of this review is that PEDOT-based bone repair materials should be understood within a fabrication–performance–translation framework. Interfacial polymerization methods, including electrochemical polymerization and VPP, are most suitable for conductive implant coatings and osseointegration interfaces. Matrix construction and crosslinking strategies are more appropriate for soft, hydrated, injectable, or drug-delivery systems. Micro-/nanoscale engineering, electrospinning, freeze drying, 3D printing, and supercritical foaming further expand PEDOTs from simple conductive coatings to spatially organized scaffolds with tunable porosity, architecture, and biological functions. Therefore, fabrication strategies should be selected according to the intended clinical application rather than being regarded as interchangeable processing approaches.

From a biological perspective, PEDOTs may regulate bone regeneration through multiple interconnected mechanisms, including charge transfer, Ca^2+^-related signaling, osteogenic differentiation, angiogenesis, immunomodulation, antibacterial activity, and electroresponsive release. However, these functions are constrained by several fundamental trade-offs, including conductivity versus degradability, mechanical robustness versus porous cell-permissive architecture, and multifunctionality versus manufacturability and reproducibility. These trade-offs explain why many PEDOT-based systems remain at the proof-of-concept stage despite encouraging in vitro and small-animal experimental results.

Moreover, the translational evaluation of PEDOTs should move beyond qualitative descriptions of nondegradability or controlled release toward quantitative assessment of degradation kinetics, conductivity retention, and multicomponent release profiles. For example, a CPM@MA conductive hydrogel containing PEDOT:PSS exhibited a mass loss of 23.82 ± 2.87% in simulated body fluid within 24 d, which increased to 55.35 ± 4.21% under ES. In the same system, the cumulative release of CaP and MgTiO_3_ reached 35.86 ± 4.18% and 37.78 ± 4.31% within 24 d, respectively, suggesting that ES can modulate both matrix degradation and inorganic component release [35]. These findings indicate that future PEDOT-based bone repair systems should report degradation rates, cumulative release profiles, conductivity retention, and biological outcomes within matched time windows to determine whether material function is synchronized with the sequential stages of bone healing.

Taken together, future research should progress from isolated material demonstrations toward route-specific design principles and standardized translational evaluation. A clinically meaningful PEDOT-based bone repair system should clearly define its target indication, conductive network stability, mechanical testing conditions, degradation and release kinetics, sterilization compatibility, animal model hierarchy, and regulatory feasibility. Establishing such a consensus will facilitate the identification of PEDOT-based strategies that are best suited for implant coatings, conformal hydrogels, porous bone scaffolds, osteochondral interfaces, or patient-specific load-bearing constructs, thereby advancing PEDOTs from promising laboratory materials to rationally designed bioelectronic platforms for bone regeneration.

## Future Prospect

Looking ahead, future research on PEDOTs for bone tissue engineering should prioritize the following aspects.

### 1. In-depth mechanistic investigation and material system design

Comprehensive investigation of the mechanisms and systematic design of PEDOT-based materials for bone repair should follow a multiscale research strategy spanning molecular to macroscopic levels. At the mechanistic level, studies should focus on molecular interfacial interactions, the relationship between micro/nanostructures and material properties, electrobiological coupling mechanisms, and degradation stability. Advanced techniques, including molecular simulations, high-resolution characterization methods, and live-cell analysis, should be employed to elucidate the fundamental mechanisms governing conductive-network formation, cellular signaling pathways, and the long-term performance evolution of these materials. In terms of material design, gradient, dynamically responsive, and biomimetic strategies should be adopted to construct multiscale composite systems that synergistically regulate structure, composition, and electrical properties. Such an approach aims to achieve multiple functionalities, including mechanical compatibility, cell guidance, and intelligent responsiveness.

**Fig. 13.**
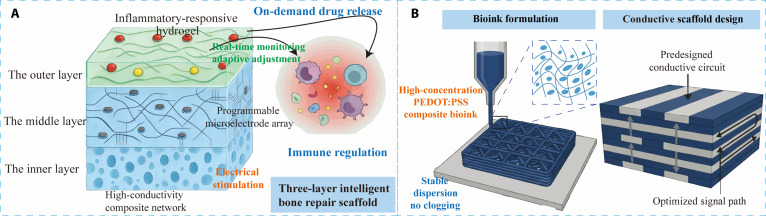
Schematic diagram of (A) the 3-layer intelligent bone repair scaffold composed of poly(3,4-ethylenedioxythiophene):polystyrene sulfonate (PEDOT:PSS) and (B) 3D printed PEDOT:PSS bioink for conductive scaffolds.

Beyond designing composite structures with gradient electrical conductivity and mechanical properties, it is also essential to integrate sensing, response, and feedback capabilities, enabling PEDOTs to dynamically regulate their behavior according to different stages of the bone repair process. For example, a 3-layer intelligent bone repair scaffold could be designed, as illustrated in Fig. 13A. The inner layer, consisting of a high-strength conductive composite network, would provide structural support and baseline ES. The middle layer, incorporating programmable microelectrode arrays, would adaptively regulate ES patterns according to real-time physiological monitoring. The outer layer, composed of an inflammation-responsive hydrogel, would enable on-demand drug release and immune regulation. This closed-loop research framework, integrating mechanistic investigation with intelligent system design, will facilitate the transition of PEDOT-based materials from conventional functional biomaterials to high-performance intelligent platforms for bone regeneration, ultimately accelerating their clinical translation.

### 2. Optimization of fabrication techniques and processing strategies

In the future, fabrication techniques for PEDOTs should evolve toward greater precision, efficiency, and reproducibility. For example, freeze drying processes can be optimized to transition from randomly distributed pores to directionally aligned porous architectures. By precisely controlling the cooling rate and freezing direction, ice-crystal growth can be guided along predefined orientations, resulting in scaffolds with highly aligned pore channels. Such architectures are more favorable for the directional growth of cells and blood vessels and are particularly suitable for repairing anisotropic tissues such as tendons, nerves, and load-bearing bone. Furthermore, the integration of artificial intelligence can facilitate the establishment of predictive models correlating freezing parameters, including cooling gradients and solvent composition, with scaffold morphology, such as pore size, porosity, and pore interconnectivity. This approach would enable inverse design while reducing the cost and time associated with empirical optimization.

Moreover, combining multiple fabrication technologies offers opportunities to optimize biomimetic structural design. For example, microprobe-assisted electrochemical polymerization enables localized and patterned deposition at the micro- or nanoscale, allowing the creation of surface microdomains with distinct chemical compositions, wettability, and electrical conductivity. Such precisely engineered interfaces can selectively regulate the behavior of different cell types, including osteoblasts and chondrocytes, thereby facilitating more sophisticated tissue regeneration. In the context of 3D printing, the development of highly concentrated and stable PEDOT:PSS-based composite bioinks is essential to ensure homogeneous dispersion during printing while preventing nozzle clogging (Fig. 13B). This strategy enables the spatial alternation or gradient distribution of conductive PEDOT phases and insulating biological phases, facilitating the construction of predefined conductive pathways within scaffolds and optimizing electrical signal transmission.

To achieve the transition from laboratory-scale samples to clinical products, the preparation process must be standardized, scalable, and capable of ensuring sterility. It is essential to establish comprehensive quality control standards covering the entire production process, including raw materials (such as batch-to-batch consistency and molecular weight of PEDOT:PSS), process parameters (such as freezing rates and crosslinker concentrations), and final product characteristics (including porosity, conductivity, and mechanical properties), together with real-time monitoring technologies (such as optical coherence tomography for real-time monitoring of the freezing process). Additionally, the use of water-based solvents, low-toxicity crosslinkers, and mild preparation conditions under low temperature and pressure should be explored to minimize environmental impact and reduce residual harmful chemicals, thereby satisfying the biosafety requirements of medical devices.

Furthermore, material compatibility with sterilization methods should be considered during the design stage to ensure that the physicochemical properties and biological activity of the materials remain unaffected after sterilization. The key to clinical translation lies in engineering integration and standardization. First, existing systems are highly sensitive to conventional clinical sterilization methods. Autoclaving may lead to hydrogel disintegration and phase separation, gamma irradiation may disrupt the conjugated backbone and result in electrical failure, whereas ultraviolet irradiation cannot penetrate the internal regions of 3D porous scaffolds. Therefore, future studies should establish mild or cold-chain sterilization protocols specifically tailored for electroactive polymers.

Beyond sterilization considerations, translational studies should clearly define the intended clinical indication and establish appropriate model hierarchies. Small calvarial defect models are valuable for evaluating osteoinductive capacity, whereas load-bearing segmental defect models, osteoporotic fracture models, infected defect models, and implant osseointegration models are required to assess mechanical reliability, immune responses, antibacterial durability, and fixation strength. Therefore, standardized reporting of model-related parameters is essential for improving comparability among studies and determining the clinical relevance of PEDOT-based bone repair systems.

### 3. Establishing a consensus evaluation framework for PEDOT-based bone repair materials

Currently, PEDOT-based studies have employed highly variable stimulation protocols, mechanical evaluations, degradation assessments, and animal models. Therefore, establishing a consensus evaluation framework within this field is necessary. Future studies should include 5 major aspects. First, material properties should be reported, including the PEDOT:PSS ratio or dopant type, solid content, molecular weight and batch information, residual oxidants or crosslinkers, and sterilization methods. Second, electrical properties should be systematically characterized, including dry and wet conductivity, impedance spectroscopy, charge storage or injection capacity, electrochemical stability, and complete electrical parameters, such as waveform, amplitude, field strength or current density, pulse width, frequency, duty cycle, stimulation duration, and electrode configuration. Third, structural and mechanical properties should be evaluated by controlling and reporting porosity, pore size, connectivity, surface roughness, expansion ratio, compressive and tensile modulus, fatigue resistance, and mechanical testing conditions under physiological humidity at 37 °C. Fourth, degradation and release behaviors should be assessed, including mass loss, PEDOT:PSS dissolution, pH variation, charge retention, release kinetics, and the synchronization of anti-inflammatory, angiogenic, and osteogenic factor release. Finally, biological evaluations should be performed using standardized cell sources and appropriate animal models, including assessments of cell viability, inflammatory markers, Ca^2+^ influx, ALP/RUNX2/OCN/SOX9 expression, mineralization, vascularization, and histomorphological changes. Establishing universal research standards will improve comparability among different laboratories and facilitate the identification of material strategies suitable for large animal studies and regulatory evaluation.

### 4. Multifunctional integration and application expansion

The future development of PEDOTs will extend beyond their current applications in bone repair toward highly integrated, multifunctional smart biomaterials with broader application scenarios. During the early stages of bone repair, infection and excessive inflammation represent major challenges. Future material designs may incorporate antibacterial components (such as Ag nanoparticles or antibiotics) or anti-inflammatory molecules (such as GA) into the material matrix. Through the engineering of pH-responsive or enzyme-triggered release mechanisms, these systems may enable intelligent release of therapeutic agents in response to infection (characterized by local pH reduction) or inflammation (indicated by increased expression of specific enzymes). This approach would enable sequential regulation by first suppressing inflammation and infection, followed by promoting tissue regeneration.

Furthermore, vascularization is critical for the repair of large bone defects because it provides essential nutrient supply. Vascular endothelial growth factors or angiogenic bioactive ions (such as Mg^2+^ or Cu^2+^) could be incorporated into PEDOT-based materials. By constructing dual-factor or multifactor sequential release systems, synergistic effects may be achieved, with early promotion of vascular ingrowth followed by guidance of osteogenic differentiation.

Taking advantage of the excellent electrochemical activity of PEDOT, these materials may also be developed into implantable biosensors. For example, PEDOT can be combined with enzymes or antibodies responsive to specific metabolites (such as glucose or lactate) or inflammatory factors (such as tumor necrosis factor-α). During material degradation or tissue regeneration in vivo, changes in electrochemical impedance or potential may occur. By enabling wireless transmission and real-time monitoring of these signals, the progression of bone healing and potential risks of early infection could be assessed noninvasively. Additionally, incorporation of piezoelectric materials (such as BaTiO_3_ or polyvinylidene fluoride) may enable scaffolds to respond to mechanical forces generated during daily physiological activities (such as muscle contraction or walking) and convert them into microcurrents. This strategy not only enables passive ES therapy but also allows the generated electrical signals to function as indicators of physiological activity [127].

The expanded application of PEDOTs requires interdisciplinary collaboration among materials science, biology, medicine, and engineering. Such integration may facilitate their application in areas including nerve regeneration and repair, cardiac tissue engineering, disease modeling, and drug screening. By applying computational materials science, interactions between PEDOT molecular chains and functional molecules (such as drugs and biological factors) can be simulated to guide the rational design of high-performance composites. Advanced 4-dimensional printing technologies may be utilized to fabricate intelligent scaffolds capable of altering their shape or function in response to environmental stimuli (such as temperature or pH), thereby enabling more dynamic and adaptive repair processes. Furthermore, artificial intelligence may assist in developing large animal models that better recapitulate human diseases, while long-term systematic evaluations of safety and efficacy performed according to clinical standards will facilitate the translation of laboratory discoveries into clinical products.

## Data Availability

No data was used for the research described in the article.
